# 3D hierarchically porous magnetic molybdenum trioxide@gold nanospheres as a nanogap-enhanced Raman scattering biosensor for SARS-CoV-2†

**DOI:** 10.1039/d1na00746g

**Published:** 2022-01-04

**Authors:** Ojodomo J. Achadu, Njemuwa Nwaji, Dongkyu Lee, Jaebeom Lee, Eser M. Akinoglu, Michael Giersig, Enoch Y. Park

**Affiliations:** Research Institute of Green Science and Technology, Shizuoka University 836 Ohya, Suruga-ku Shizuoka 422-8529 Japan park.enoch@shizuoka.ac.jp ojodomo.john.achadu@shizuoka.ac.jp +81-54-238-4887 +81-54-238-3306; International Academy of Optoelectronics at Zhaoqing, South China Normal University Liyuan Street 526238 Guangdong China njemuwa.nwaji@zq-scnu.org; Dept. of Chemistry, College of Natural Science, Chungnam National University 99 Daehak-ro, Yuseong-gu Daejeon 34134 Korea; Institute of Fundamental Technological Research, Polish Academy of Sciences 02-106 Warsaw Poland; Laboratory of Biotechnology, Department of Bioscience, Graduate School of Science and Technology, Shizuoka University 836 Ohya, Suruga-ku Shizuoka 422-8529 Japan; International Institute for Nanocomposites Manufacturing, WMG, University of Warwick CV4 7AL Coventry UK

## Abstract

The global pandemic of COVID-19 is an example of how quickly a disease-causing virus can take root and threaten our civilization. Nowadays, ultrasensitive and rapid detection of contagious pathogens is in high demand. Here, we present a novel hierarchically porous 3-dimensional magnetic molybdenum trioxide–polydopamine-gold functionalized nanosphere (3D mag-MoO_3_–PDA@Au NS) composed of plasmonic, semiconductor, and magnetic nanoparticles as a multifunctional nanosculptured hybrid. Based on the synthesized 3D mag-MoO_3_–PDA@Au NS, a universal “plug and play” biosensor for pathogens is proposed. Specifically, a magnetically-induced nanogap-enhanced Raman scattering (MINERS) detection platform was developed using the 3D nanostructure. Through a magnetic actuation process, the MINERS system overcomes Raman signal stability and reproducibility challenges for the ultrasensitive detection of SARS-CoV-2 spike protein over a wide dynamic range up to a detection limit of 10^−15^ g mL^−1^. The proposed MINERS platform will facilitate the broader use of Raman spectroscopy as a powerful analytical detection tool in diverse fields.

## Abstract 2

A multifunctional 3D magnetic molybdenum trioxide@gold nanosphere is deployed as a plug and play biosensor for SARS-CoV-2 spike proteins *via* ACE2-mediated immunocapture and magnetic-induced nanogap-enhanced Raman scattering (MINERS).

## Graphical abstract

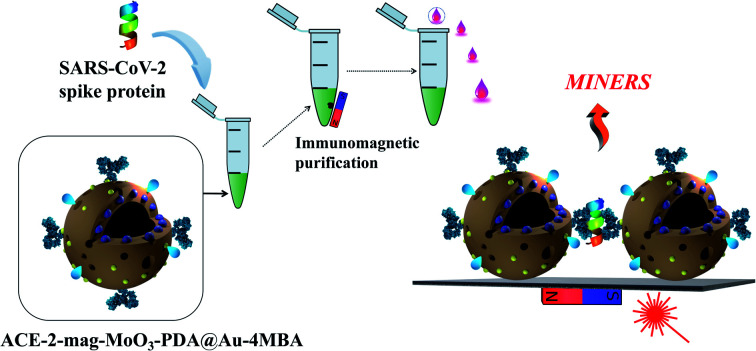

## Introduction

The development of powerful surface-enhanced Raman spectroscopy (SERS)-based biosensors for pathogens detection remains challenging, requiring integrated and continuous research efforts. The ultimate goal is to develop material substrates and/or processes with exceptional qualities to generate reproducible and stable Raman signals.^1,2^ With this in mind, metal Au and Ag nanoparticles (NPs) with sophisticated morphologies and dimensions, such as cubic, octahedral, and star-shaped nanostructures, have been introduced into SERS detection technology.^3–5^ This is due to the propensity of nanostructures with complex morphologies to resonantly couple to light with an intense scattering at the nanoscale, resulting in increased electromagnetic (EM) field strength for “hotspots” generation.^6,7^ Meanwhile, the state-of-the-art plasmonic Au or Ag NPs face competition from metal oxide semiconductor nanostructures in SERS technology due to their competitive performance and ability to function as both plasmonic and electroactive semiconductor materials.^8–10^ Therefore, the development of innovative SERS-based biosensors for reliable biological assays using conventional plasmonic metal NPs is slowly eroding due to contentious stability, biocompatibility, and reproducibility.^11–16^ Besides, their high costs further limit their practical use. In these situations, multi-component hybrid materials of plasmonic metal NPs and other functional materials are explored in the search for ideal SERS substrates. The composition of the hybrid nanostructures is an essential necessity in this pursuit.^12,17–19^

Recently, metal oxide semiconductors of titanium oxide (TiO_2_), tungsten oxide (WoO_*x*_), and molybdenum oxide (MoO_*x*_) are highlighted as excellent candidates with promising SERS performances.^14,20–25^ The SERS properties of metal oxides are directly attributed to a photo-induced charge-transfer (PICT) mechanism, in addition to the surface polarization effect caused by the oxide defect states and molecular polarizability to enhance the Raman signals of interacting reporters.^14,24,26,27^ Therefore, the hybrids of metal oxides with plasmonic metal NPs are predicted to give significantly superior performance compared to using metal oxides or plasmonic metal NPs separately. The advantage of custom-designed composites of metal oxide-plasmonic NPs mentioned above is that the semiconducting oxides are most chemically stable and have excellent electron transport systems for SERS through chemical mechanism.^11,12,14^ At the same time, plasmonic NPs most efficiently and predominantly contribute to SERS through EM field enhancement.^4,28^ Therefore, preparing innovative hybrid nanostructures of metal oxide-plasmonic NPs with distinct properties while integrating the best of both materials will result in excellent SERS substrates that can generate high-throughput and reliable analytical data. Besides, in SERS diagnostic-oriented biosensor nanotechnology, introducing magnetic functionality into high-performance optical materials creates magneto-optical effects,^29–31^ making it possible to fine-tune SERS substrates' interparticle distance for optimal distribution of “hotspots” and/or charge transport *via* magnetic actuation.^32–34^ In addition, magnetic functions can be used to rapidly separate and enrich bio-targets from complex samples, thereby eliminating substrate interference^35–37^ and significantly improving the sensitivity of biological assays.

In this work, we present a multifunctional 3D nanostructure consisting of metal oxide, plasmonic, and magnetic NPs as a dual-mechanistic SERS substrate and propose a new approach for their use in the design of an ultrasensitive, reproducible, and reliable biosensing platform using Raman spectroscopy. By explicitly using a wet chemical route, we synthesized hierarchically porous 3D magnetic molybdenum trioxide–polydopamine@Au nanospheres (mag-MoO_3_–PDA@Au NS), which exhibits high chemical stability and magnetic strength with a polydopamine (PDA) 3D nanoskeleton. The optical properties of the 3D mag-MoO_3_–PDA@Au NS displayed high SERS activities of Raman probes adsorbed on their surface to confirm the functional applicability of the 3D nanostructure. The observed Raman signal enhancements are attributed to the amplified charge-transfer effect synergistically coupled with localized surface plasmons contributed by the MoO_3_ and AuNPs components of the 3D nanostructure,^8,12,17,19^ respectively. Interestingly, the optical enhancing activity of the 3D nanostructure studied under the influence of an external magnetic field resulted in unprecedented Raman signal amplification, which is related to a magneto-optical actuation phenomenon.^30–32^ After careful investigation and optimization protocols, a magnetically-induced nanogap-enhanced Raman scattering (MINERS) process was developed to implement a high-performance biosensing technology for the rapid diagnosis of COVID-19 *via* the spike proteins of SARS-CoV-2.

## Results and discussion

### Synthesis and characterization of 3D hierarchical mag-MoO_3_–PDA@Au NS

**Fig. 1:**
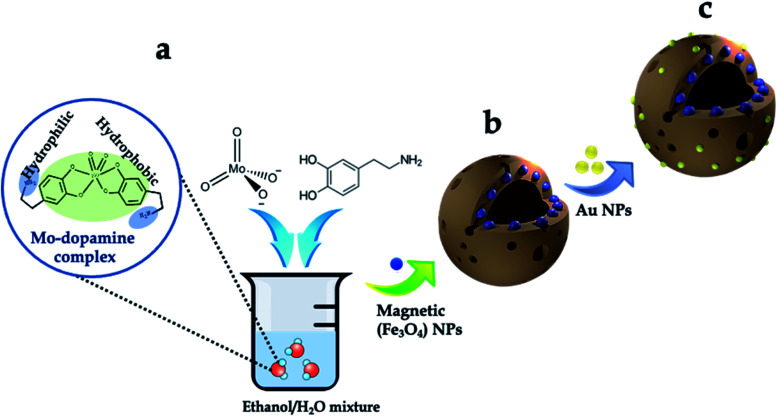
Synthesis of the 3D hierarchical and porous mag-MoO_3_–PDA@Au nanostructure. (a) Schematic illustration of the synthesis of MoO_3_–PDA nanostructure. (b) *In situ* encapsulation of magnetic nanoparticles to derive mag-MoO_3_–PDA. (c) Hierarchical surface functionalization with Au NPs.

The synthesis scheme of the hierarchically porous 3D mag-MoO_3_–PDA@Au NS is shown in Fig. 1. To prepare this 3D hollow and porous nanostructure, chemically active ammonium heptamolybdate [(NH_4_)_6_Mo_7_O_24_] and dopamine were used as precursors. The specific formation mechanism first involves the chelation of the Mo_7_O_24_^6−^ anion to the catechol-rich group of the organic dopamine to form a sparsely water-soluble Mo–dopamine complex as depicted in Fig. 1a. By adding an ethanolic solution to the reaction medium, the polymer complex (PDA) with water nanobubbles was formed in the presence of both hydrophilic and hydrophobic units at a water–oil interface. Then, an interfacial interaction causes the hydrophobic group of the Mo–dopamine complex to direct towards the ethanol phase with a corresponding orientation of the hydrophilic groups to the aqueous phase while a few drops of NH_4_OH induced autopolymerization to form the Mo–PDA complex. Under these conditions, the eccentric isotropic growth of PDA in its usual 2D pattern is radically inhibited by the Mo complexation.^38,39^ This rather initiates an interfacial polymerization of the hydrophobic Mo–dopamine around the nanobubbles in the aqueous phase to form hollow nanospheres with unique porosity and a hierarchical 3D morphology. To magnetize the 3D nanostructure, magnetic NPs (∼10 nm) were trapped within the pores of the Mo–PDA complex *via* Fe ions chelation to PDA^40^ during the polymerization process, as depicted in Fig. 1b. Unlike our synthesis route, the integration of different precursor components to form a single nanostructure usually proceeds in a multi-step process which is often limited by the compatibility of the components. Our synthetic approach uses only a one-step strategy to integrate different precursors to create hierarchical nanostructures, thus making it superior and straightforward. Finally, we obtained our robust and multifunctional hierarchical mag-MoO_3_–PDA@Au NS, with dual plasmonic function, using the amine moieties of the mag-MoO_3_–PDA derivative to decorate their surfaces with AuNPs of ∼20 nm (Fig. 1c). This relies on the strong interaction of AuNPs with the amine functional groups^41,42^ of the mag-MoO_3_–PDA.

**Fig. 2:**
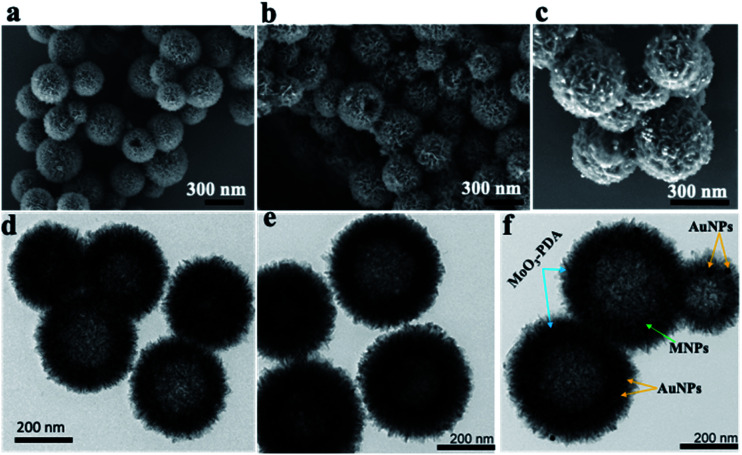
Morphology and structural characterization of the various 3D nanostructures. SEM images of (a) MoO_3_–PDA NS; (b) mag-MoO_3_–PDA NS, and (c) mag-MoO_3_–PDA@Au. Their corresponding TEM images are shown for (d) MoO_3_–PDA NS, (e) mag-MoO_3_–PDA NS, and (f) mag-MoO_3_–PDA@Au NS.

The morphology and nanostructure of the hierarchical mag-MoO_3_–PDA@Au NS were elucidated using various techniques, from electron microscopy to spectroscopy. First, the scanning electron microscopy (SEM) results are shown in Fig. 2a–c. Morphologically, the SEM results confirmed the hollowed and porous nanostructures starting from MoO_3_–PDA (Fig. 2a). These porous nanostructures were uniformly preserved along the modification pathway to obtain a magnetic derivative mag-MoO_3_–PDA (Fig. 2b) and the hierarchical mag-MoO_3_–PDA@Au NS (Fig. 2c).

Overall, the SEM images showed well-defined hierarchical nanostructures with average diameters typically in the range of 200–400 nm. Notably, the 3D mag-MoO_3_–PDA@Au NS of interest displayed the grafting of plasmonic AuNPs on their surface to impart some surface roughness, as seen in Fig. 2c, while maintaining the 3D morphology. All these features are poised to benefit the intensity and uniformity of the SERS properties of the prepared dual-plasmonic 3D nanostructure. To confirm the incorporation of magnetic NPs into the pores of the nanostructure, N_2_ sorption isotherms (ESI Fig. S1†) showed that the specific surface area of mag-MoO_3_–PDA is 217.30 m^2^ g^−1^, which is greater than 200.92 m^2^ g^−1^ observed for MoO_3_–PDA. It indicates that the magnetic NPs were not only incorporated into the hierarchical structure but also slightly increased the size as observed in the SEM results, which may have resulted from the magnetic NPs deposition on the surface of MoO_3_–PDA. Like SEM images, the related nanostructures' images observed by transmission electron microscopy (TEM) clearly emphasized the porous morphologies (Fig. 2d–f). Since the magnetic NPs are buried in the porous nanoskeletons, they are not discernible in the TEM image in Fig. 2e. The TEM image of mag-MoO_3_–PDA@Au NS in Fig. 2f clearly shows AuNPs spotted on the surface of the 3D nanostructure, which confirms the results of the SEM image in Fig. 2c.

**Fig. 3:**
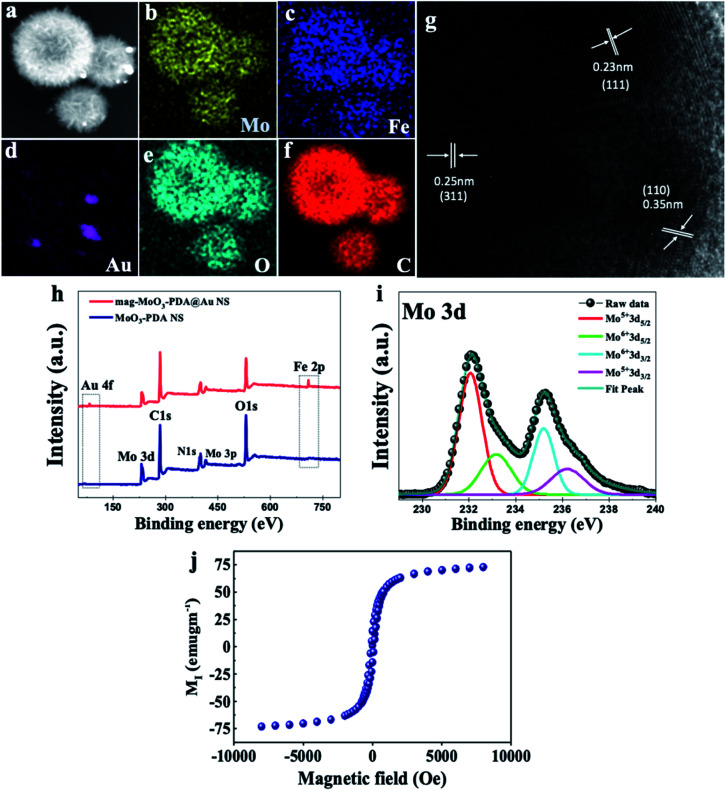
Elemental mapping, HR-TEM, and XPS characterization of the 3D mag-MoO_3_–PDA@Au nanostructure. (a) SEM; (b–f) EDS mapping. (g) HR-TEM image. (h) XPS survey scan. (i) High-resolution Mo 3d XPS spectrum. (j) Saturation magnetization.

The presence of different types of atomic species was confirmed by the energy dispersive spectroscopy (EDS) mapping results of the nanostructure in Fig. 3a, which clearly show the uniform distribution of Mo, Fe, Au, O, and C (Fig. 3b–f), demonstrating the quality of the prepared 3D nanostructure. The EDS elemental mapping of mag-MoO_3_–PDA NS (without AuNPs assemblies) is shown in ESI Fig. S2.† The high-resolution TEM (HR-TEM) analysis of the 3D mag-MoO_3_–PDA@Au NS clearly revealed three distinctive adjacent crystal planes of (110), (311), and (111) corresponding to MoO_3_, magnetic (Fe_3_O_4_), and AuNPs, respectively.^14,29,43^ These correspond to the lattice fringes of 0.35, 0.24, and 0.23 nm deduced from the HR-TEM image (Fig. 3g). The HR-TEM result further supports the EDS data for the qualitative presence of key components that impart both plasmonic and magnetic properties to the 3D nanostructure. To complement the EDS mapping and HR-TEM results, an X-ray photoemission spectroscopy (XPS) analysis was performed to determine the mixed chemical states of the 3D nanostructure and the definitive presence of the key components revealed by EDS elemental mapping. In Fig. 3h, the XPS wide scan of MoO_3_–PDA NS (blue spectrum) shows the characteristic peaks of Mo 3d, C 1s, Mo 3p, O 1s, and N 1s. In addition to the elements mentioned above, the XPS wide scan of mag-MoO_3_–PDA@Au NS (red spectrum) shows additional peaks at 83.9 eV and 706.8 eV, attributed to Au 4f and Fe 2p, respectively.^3,35^ The Gaussian-fitted high-resolution XPS results are further presented in Fig. 3i. The strong intensity peaks with binding energies of 231.6 eV and 235.0 eV result from the Mo 3d orbital splitting (Mo 3d_5/2_ and Mo 3d_3/2_) of the hexavalent Mo^6+^ state.^17,44^ The observed peaks at 233.2 eV and 236.1 eV can be attributed to the pentavalent and tetravalent states (Mo^5+^/Mo^4+^) generated from the oxidation states of MoO_3_.^45,46^ The binding energies at 398.1 eV and 401.8 eV can be assigned to the secondary and primary amines of the PDA component of the 3D nanostructures, making it possible to couple AuNPs as confirmed by the appearance of the peak at the binding energy of 397.1 eV due to the N–Au bond.^47,48^ An examination of the high-resolution Au 4f peaks of the mag-MoO_3_–PDA@Au NS, ESI Fig. S3,† showed that the Au_5/2_ and Au_7/2_ peaks have spin–orbit asymmetry at 86.1 eV and 84.4 eV, respectively. Meanwhile, it is known that sulfur or nitrogen attachment to the surface of AuNPs exhibits higher binding energies in XPS compared with bulk metals.^49^ Therefore, the peak observed at the binding energy of 89.3 eV corresponds to AuNPs binding to the amine (N) of the PDA to clarify the anchor site of AuNPs to the mag-MoO_3_–PDA. The high-resolution XPS of Fe 2p in ESI Fig. S4† displayed a noisy signal and background that obscures the Fe 2p_3/2_ or Fe 2p_1/2_ peaks, confirming the capture of magnetic NPs inside the nanopores of the 3D nanostructure. To gain further insights into the magnetic functionality of the 3D mag-MoO_3_–PDA@Au NS, the saturation magnetization was measured, and shows a high magnetic moment value (at 300 K) with a remanence effect of ∼76.6 emu g^−1^ according to the magnetic hysteresis curve in Fig. 3j. This indicates the ferromagnetic properties of the nanostructure's magnetic component. The strong magnetic moment of the 3D mag-MoO_3_–PDA@Au NS provides a special magnetic function to effectively tune the nanostructural gaps between its dimers and trimers under the influence of external magnetic fields,^35^ as well as for biological separation of targets from complex samples.

Finally, the structural characterization of the 3D mag-MoO_3_–PDA@Au NS proceeded with the elucidation of the crystalline phase of the MoO_3_–PDA NS using X-ray diffractometry (XRD) (ESI Fig. S5†). The XRD patterns recorded for MoO_3_–PDA and other derivatives show the indexation of the nanostructures to the monoclinic phase of MoO_3_ (JCPDS no. 05-0508),^14^ indicating that the principal component of the 3D nanostructures is MoO_3_. The broadly strong peaks around 2*θ* = 13.9–16.7° are attributed to the MoO_3_–PDA complex formed between the Mo_7_O_24_^6−^ anion and hydroxyl (catechol) groups of dopamine.^50^ For the magnetic NPs derived nanostructure (mag-MoO_3_–PDA), no further peak broadening was observed after incorporating the magnetic NPs (ESI Fig. S5†), indicating that the magnetic NPs' uniform size was maintained upon grafting on the surface and/or within the nanopores of the MoO_3_–PDA. Therefore, the characteristic diffraction peaks of magnetic NPs are not strong in the mag-MoO_3_–PDA NS; however, the small broad peak around 32.5° corresponds to (220) planes of Fe_3_O_4_ (JCPDS no. 89-0691).^51^ In the 3D mag-MoO_3_–PDA@Au NS (ESI Fig. S5†), the strong peak at 39.05 and 44.5° matched the (111) and (200) crystallographic planes of Au (JCPDS no. 04-0784),^52^ which further confirmed the decoration of the 3D nanostructure with AuNPs. These detailed characterization results demonstrate the successful preparation of the 3D hierarchically porous and hollow mag-MoO_3_–PDA@Au NS through a simple approach and, most importantly, at ambient temperature. ESI discussion and data on the characteristic UV-Vis properties of our 3D mag-MoO_3_–PDA@Au NS and precursor materials are provided in the ESI (Note 1 and Fig. S6†).

### Raman scattering activity and dual-enhancement mechanisms

**Fig. 4:**
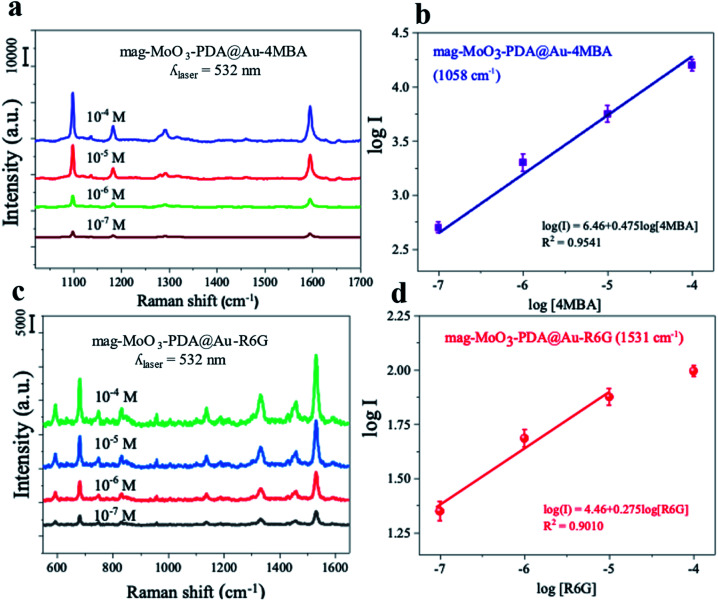
SERS properties of 3D mag-MoO_3_–PDA@Au nanostructure (0.1 g mL^−1^). (a and c) Raman spectra of 10^−7^–10^−4^ M 4-MBA or R6G obtained using the 3D mag-MoO_3_–PDA@Au NS as a SERS substrate. (b and d) SERS intensity of 4-MBA or R6G at 1054 cm^−1^ or 687 cm^−1^ as a function of different concentrations.

To investigate the Raman enhancement activity of the 3D mag-MoO_3_–PDA@Au NS, specific Raman reporters such as 4-mercaptobenzoic acid (4-MBA) and rhodamine 6G (R6G) were adsorbed on the surface of the mag-MoO_3_–PDA@Au NS, and their catalyzed SERS activities were evaluated. In Fig. 4, the characteristic Raman peaks of 4-MBA or R6G molecules were enhanced, demonstrating the strong SERS properties of the 3D mag-MoO_3_–PDA@Au NS. In addition, quantitative relationships between the Raman reporters and the 3D mag-MoO_3_–PDA@Au NS could be observed. By performing SERS measurements at resonance excitation of 532 nm with different concentrations (10^−7^ to 10^−4^ M) of 4-MBA (Fig. 4a and b) or R6G (Fig. 4c and d), respectively, the SERS signals were markedly enhanced compared to the reporter molecules alone. This level of SERS effect at a low concentration of 10^−7^ M in each reporter molecule suggests the extremely high sensitivity of the 3D mag-MoO_3_–PDA@Au NS as a SERS substrate. This is reasonably due to the EM and/or chemical enhancement mechanisms possessed by the 3D mag-MoO_3_–PDA@Au NS resulting from the constitutive binary plasmonic and semiconductor components of the nanostructure.^21,53^

Furthermore, the SERS enhancement factor (EF) (calculation details in ESI Note 2†), evaluated using the strongly enhanced peaks at 1058 cm^−1^ for 4-MBA or 656 cm^−1^ for R6G, were used as a measure to assess the comparable or superior capacity of the 3D mag-MoO_3_–PDA@Au NS as a SERS substrate. Meanwhile, the distinctive output index of the 3D mag-MoO_3_–PDA@Au NS, as shown in ESI Table S1,† was the outstanding overall EF values of 7.6 × 10^9^ and 6.5 × 10^7^ recorded for 4-MBA and R6G, respectively, thanks to binary components and 3D morphology of the nanostructure.

In particular, the semiconductor oxide MoO_3_ facilitates a high charge-transfer efficiency,^14,23,54^ which dramatically increases the Raman scattering cross-section of the 3D nanostructure. This is complemented by the classical localized surface plasmon provided by AuNPs roughening the surface of the 3D nanostructure. To emphasize this point, ESI Table S1† provides a detailed evaluation of the EF of 4-MBA or R6G with 3D mag-MoO_3_–PDA@Au NS compared to EF obtained in mag-MoO_3_–PDA NS or MoO_3_–PDA. From the difference in EF values, it is clear that different kinds of enhancement mechanisms are at work, or at least the same mechanisms are not engaged equally or similarly. Thus, there is no doubt that the as-synthesized 3D mag-MoO_3_–PDA NS is an excellent SERS substrate. This is based on a dominating charge-transfer mechanism induced by oxygen defects in MoO_3_ semiconductor oxide and a plasmon resonance effect.^8,45^ Besides, the optical properties have been reinforced by AuNPs speckled on the surface of the 3D mag-MoO_3_–PDA NS, which offers the prospect of a plasmon-enhanced Raman scattering effect coupled with charge transfer. The experimental results and calculated EFs of the 4-MBA or R6G convincingly support the expected occurrence of various types of Raman scattering mechanisms in the 3D mag-MoO_3_–PDA@Au NS.

The observed difference in the SERS EF for 4-MBA or R6G requires some discussion. Unlike R6G, thiolated 4-MBA can easily bind to the surface of the 3D mag-MoO_3_–PDA@Au NS through a strong Au–S interaction.^55^ This binding interaction is likely to result in a significant Raman enhancement in 4-MBA as observed in the higher EF and sensitivity of 4-MBA adsorbed on the 3D mag-MoO_3_–PDA@Au NS. Moreover, as variables such as the substrate and/or molecule size, background noise, and resonance Raman mode can all affect Raman reporters' SERS signals,^1,56^ we obtained robust data by comparing results from resonant and non-resonant modes using the 532 and 785 nm excitations, respectively. Further experiments were then narrowed down using 532 nm excitation specifically due to the resonance absorptions of R6G or 4-MBA at this wavelength. The optimized results indicate that the resonant Raman effect at 532 nm plays a critical role in the Raman enhancement observed in the 3D mag-MoO_3_–PDA@Au NS. Details of the SERS spectra at 785 nm laser excitation can be found in the ESI data.†

**Fig. 5:**
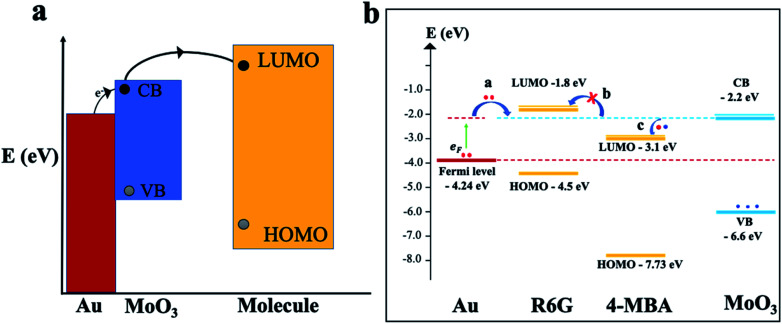
PICT schematics and DFT. (a) Schematic illustration of PICT process of the 3D mag-MoO_3_–PDA@Au NS. (b) Conclusion of calculated results and the possible PICT mechanism and energy-level diagram of 4-MBA or R6G and 3D mag-MoO_3_–PDA@Au NS.

To gain further insights and a solid understanding of the enhancement mechanisms at play between the 3D mag-MoO_3_–PDA@Au NS and 4-MBA or R6G, a related matching degree of the calculated energy level of the 3D mag-MoO_3_–PDA@Au NS and the molecular orbital alignments of 4-MBA or R6G need to be elucidated. To clarify this, theoretical calculations based on density functional theory (DFT) were employed. The DFT results and energy-level diagrams in Fig. 5a and b schematically illustrate the possible photo-induced charge transfer (PICT) pathways for the mag-MoO_3_–PDA@Au-4-MBA and mag-MoO_3_–PDA@Au-R6G hybrid states, respectively. To explain the PICT pathways and to confirm that our speculation about the dual-SERS enhancement mechanisms is consistent with our experimental results, we specifically projected the electronic structure of our 3D mag-MoO_3_–PDA@Au NS using DFT and evaluated possible new electronic states at the MoO_3_–PDA and Au interface. For this purpose, the Au Fermi level, conduction band (CB), and valence band (VB) of the 3D mag-MoO_3_–PDA@Au NS were calculated, as well as the HOMO and LUMO levels of 4-MBA and R6G. As represented in Fig. 5b, the energy barrier between the Fermi level of Au and the LUMO of 4-MBA in the mag-MoO_3_–PDA@Au-4-MBA system is 1.14 eV, which indicates the possibility of a direct electron transfer under photo-excitation of 532 nm (2.33 eV) or 785 nm (1.58 eV).^57^

Meanwhile, considering the defects engineered SERS properties of semiconductor oxide nanostructures,^10,11,14,22^ we hypothesize that the oxygen defect states of MoO_3_ within the hybrid systems can experience catalyzed charge redistribution *via* Au-assisted electrons injection.^58,59^ Consequently, the semiconductor MoO_3_ band edges at the interfaces with 4-MBA or R6G become populated, leading to energy-level realignment due to photoexcited Fermi electrons (EF) transfer to the CB of MoO_3_ (Fig. 5b).^58^ In this scenario, the energy barriers between the Fermi level and the CB of MoO_3_; the LUMO of 4-MBA and CB of MoO_3_ are significantly narrowed from 2.04 and 1.14 eV to 0.44 and 0.7 eV, respectively. Thus, PICT can easily occur in the mag-MoO_3_–PDA@Au-4-MBA hybrid system. Conversely, R6G's LUMO lies higher at −1.8 eV than the CB of MoO_3_ at −2.2 eV, meaning there is a rare likelihood of a charge transfer to occur between R6G and mag-MoO_3_–PDA@Au NS (Fig. 5b). Overall, the DFT calculations elucidate the PICT potential, at equilibrium, between 4-MBA or R6G and the 3D mag-MoO_3_–PDA@Au NS substrate to provide theoretical evidence to explain the experimental Raman enhancements observed in the 3D mag-MoO_3_–PDA@Au NS.

### MINERS “hotspots” engineering process

**Fig. 6:**
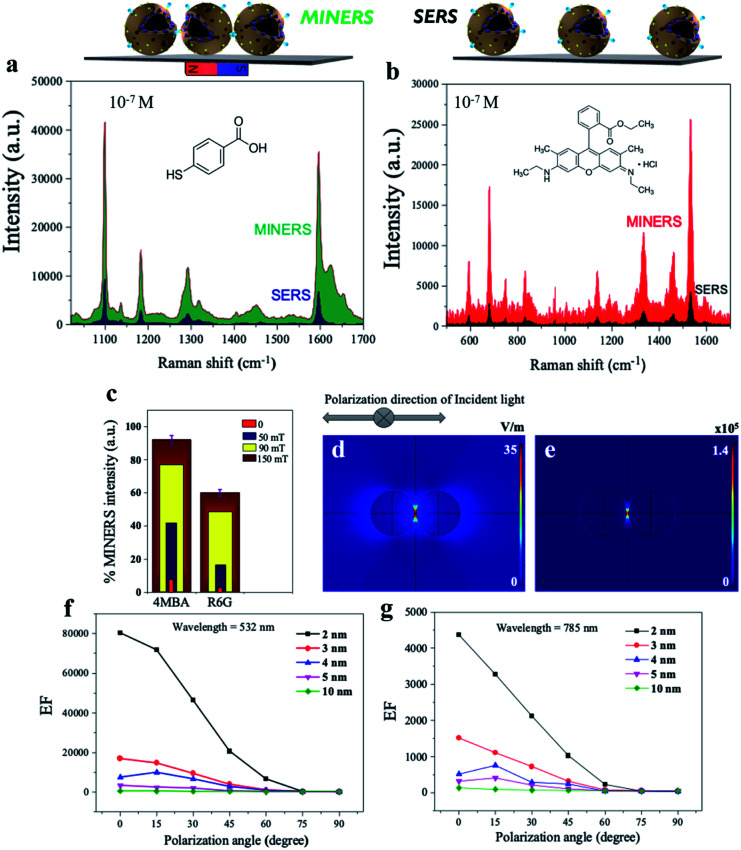
MINERS “hotspots” engineering concept in the 3D mag-MoO_3_–PDA@Au nanostructure and finite element method (FEM) simulation. (a and b) Comparison of the MINERS and SERS spectra of 10^−7^ M 4-MBA or R6G obtained using the 3D mag-MoO_3_–PDA@Au NS under an applied external magnetic field of 150 mT. (c) MINERS spectra intensity at different magnetic field power. (d and e) FEM simulated EM field distributions (in air medium, *λ* = 532 nm, *E*_0_ = 1 V m^−1^) and the EF of the 3D mag-MoO_3_–PDA@Au dimers at 2 nm nanogaps. (f and g) Numerically simulated EF values in the 3D mag-MoO_3_–PDA@Au NS dimers showing calculated EF *versus* different nanogaps (2–10 nm).

Several strategies are being developed continuously to increase the strength and viability of plasmonic “hotspots” to generate robust SERS substrates. One of these strategies is the colloidal self-assembly of NPs to conserve “hotspots” configurations by using various functions and parameters from physicochemical and/or mechanical processes.^60–63^ There is now much scientific evidence that the plasmonic “hotspots” for Raman scattering can be further strengthened by the colloidal interactions of adjacent pairs of nanoparticles or their aggregates.^61,64^ In addition, the interference of plasmonic excitations caused by the scatterings from interparticle junctions and/or surfaces can also provide the most spatially delocalized areas with strong EM fields for catalyzed high-intensity SERS signals.^5,64–66^ Considering that our synthesized 3D mag-MoO_3_–PDA@Au NS possesses excellent magnetization properties and can be tuned and self-assembled by applying an external magnetic field, we investigated the Raman scattering activities under the influence of a magnetic field. An external magnetic force is used to modulate the interparticle spacing and/or intermolecular junctions of the 3D nanostructure in dimeric or multimeric configurations. Fig. 6a and b shows the results of the advanced hypersensitive SERS process, termed MINERS in this work, which takes advantage of the magnetic properties of our 3D mag-MoO_3_–PDA@Au NS to reversibly tune the Raman scattering cross-sections of the molecular probes, 4-MBA and R6G, respectively. The adsorbed molecules interposed between adjacent pairs of the 3D nanostructure experience an increase in MINER signal intensity by modulating the size of the interparticle nanogaps at different magnetic field strengths (Fig. 6c).

Therefore, the MINERS signals were collected at the interparticle nanogap “hotspots” or junctions from the stoichiometrically controlled dimers or trimers of the 3D mag-MoO_3_–PDA@Au NS-4-MBA or R6G systems and also for their uncontrolled aggregates. Compared to the conventional SERS process, the signals generated by the MINERS process are strikingly more intense at extended magnetic field intensities, as shown in Fig. 6a and b for 4-MBA and R6G systems. At 150 mT magnetic field, the MINERS signals increased in the order of 10^4^ or 10^2^ magnitudes for the 4-MBA or R6G hybrids, respectively, compared to the SERS signals (detail in ESI Table S2†). This work's MINERS data and results exhibit a high degree of repeatability, good signal-to-noise ratio, and qualitative differentiation outputs. In addition to the magnetic properties that underpin the MINERS “hotspot” engineering concept, our nanostructure's bulky, hollow, and porous morphology is considered a special advantage. This provides a greater capacity for plasmonic “hotspots” clusters through a volume-enhanced Raman scattering effect as well as the efficient adsorption of Raman molecules and/or target analytes with significantly improved incident light capture capacity.^64,67^ Due to these properties, our developed MINERS approach effortlessly facilitated the generation of ultra-enhanced mag-MoO_3_–PDA@Au NS-based Raman scattering signals by strongly converging nearby nanostructures reversibly in a magnetic field. To better understand the “hotspots” formation as a function of the close contact and/or the nano-gap size modulation resulting from the “pushing together” of the 3D mag-MoO_3_–PDA@Au NS by an applied magnetic force, we performed numerical simulations of the distribution of the local electric field (|*E*|^4^) around the dimers using a spherical geometry and parameters approximating our 3D nanostructure. Because of the fundamentality of using an appropriate plasmonic/laser resonance excitation to yield optimal signal efficiency, the local EM field strength dynamics were also calculated at 532 and 785 nm, respectively.

The rationalized Mie theory^15,59^ can be used to compute the spatial distribution of EF at “hotspots” between spherical dimers of the nanostructure using the framework of the |*E*|^4^ approximation.^15,17^ The values of |*E*|^4^ and EF of the nanogap “hotspots” existing in the dimer of the nanostructure reach a maximum of ∼10^5^ for an interparticle distance of 2 nm (Fig. 6d and e), at 532 nm excitation (direction of polarization, 0°). This indicates that the horizontal alignment of the dimers along the direction of polarization of the EM field is more favourable. Further details of different direction of polarization of the EM fields (15–90°) simulation results are provided in ESI Fig. S7 and S8.† Overall, the highest EF value could be obtained at 532 nm excitation (direction of polarization, 0°), with nanogap sizes less than 10 nm capable of providing significant EFs, as shown by the simplified plots of simulated EF values as a function of interparticle distance in Fig. 6f and g. Taken together, the numerical computational results allow us to reasonably substantiate the signal amplification observed in our MINERS strategy under increasing magnetic field strengths (Fig. 6c). This results in the formation of unique nanogap-induced electromagnetic “hotspots” by maintaining a minimum distance between the rough surfaces of adjacent assembles of the 3D nanostructure under the application of an external magnetic field. However, we believe that in reality, and as observed in our experimental results, the |*E*|^4^ and EF values could be several orders of magnitude larger than the simulated results due to some inadvertent data or parameters inputs. In addition, because it is well known that most Raman scattering signals come from only a small fraction of molecules located in regions of high-intensity EM fields,^63,67^ a complete theory and understanding of the actual mechanisms of our MINERS approach would require advanced quantum plasmonic and mechanical calculations, which is of future interest.

### MINERS bioassays of SARS-CoV-2 spike protein

**Fig. 7:**
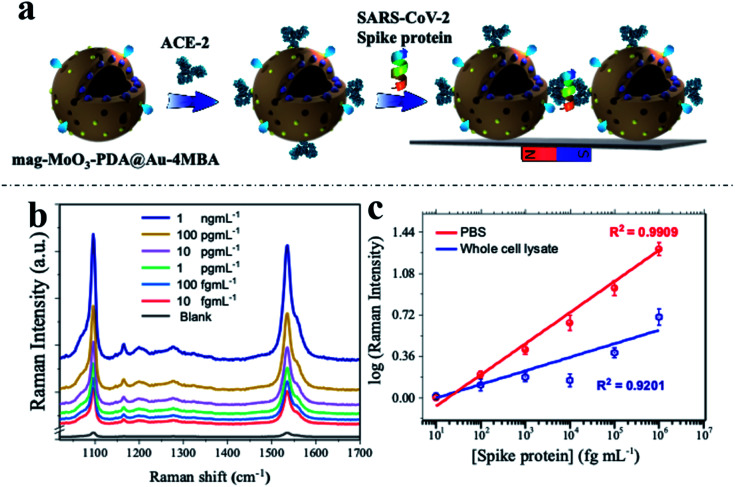
MINERS bioassay of SARS-CoV-2 spike protein. (a) Schematics of the 3D mag-MoO_3_–PDA@Au nanostructure functionalization with ACE-2 and capture/immunoreaction with spike protein in MINERS substrate. (b) MINERS spectra generated in the presence of different concentration of SARS-CoV-2 spike protein. (c) Corresponding calibration plots for MINERS bioassay of SARS-CoV-2 spike protein in PBS and whole cell lysate media.

To demonstrate the real-world applicability of our proposed MINERS technique, we have extended our MINERS “hotspots” engineering approach for the biosensing of SARS-CoV-2 spike protein using the specific angiotensin-converting enzyme 2 (ACE-2) as the biological capture agent for the spike protein target.^68^ To do so, we deployed the use of a sandwich-type immunoreaction, with the spike protein of SARS-CoV-2 serving as a linker between our ACE2-modified 3D nanostructure dimers, thus manipulating their nanogap size for the robust biosensing of the mediating immuno-sandwiched SARS-CoV-2 spike protein as depicted in Fig. 7a. Since the SARS-CoV-2 spike protein uses ACE2 as a receptor for entry into target cells,^69,70^ the spike protein has become the primary antigen for COVID-19 biosensors development.^71,72^ Meanwhile, as mutations in the gene encoding the spike protein are constantly being discovered,^73,74^ further development of biosensors that closely evaluate and monitor the antigenic mutations of the spike protein of the raging virus has clearly become critical. In this regard, our MINERS biosensor could be adopted as an ultrasensitive Raman-based biosensing technology that does not diverge significantly in response to different strains or mutations of the SARS-CoV-2 spike protein and would be highly beneficial at this time.

The developed protocol of the MINERS biosensor involves the initial biosynthesis of an active immunocomplex of ACE2 and mag-MoO_3_–PDA@Au-4-MBA system for the rapid binding of SARS-CoV-2 spike protein (Fig. 7a). This was followed by the stoichiometric control of the formation of multimeric species of the formed ACE2-mag-MoO_3_–PDA@Au-4-MBA immunocomplex for nanogap-derived multiple immunocomplex reactions in the presence of spike protein.

Using our optimized MINERS protocol and a combination of strategies involving the careful control of stoichiometric amounts of spike protein and anti-ACE2 to inhibit and regulate the reactivity of ACE2 to SARS-CoV-2 spike protein, we were able to create different nanogaps between the spike protein-mediated-ACE2-mag-MoO_3_–PDA@Au-4-MBA immunocomplexes and probably formed different multimers in the process for modulated “hotspots” intensity. Besides, the MINERS protocol ensured the magnetic enrichment and convergence of the immunocomplexed 3D nanostructure in a magnetic field to firmly close the nanogaps and induced significantly enhanced MINERS signals of the 4-MBA reporter as a function of the concentration of the target spike protein. The results in Fig. 7b and c show that the SARS-CoV-2 spike protein can be detected in the fg mL^−1^ range with a wide linear dynamic detection range from 10 fg mL^−1^ to 1 ng mL^−1^. Specifically, the MINERS signalling responses are cascaded according to the concentration of the spike protein, suggesting that additional ACE2-mag-MoO_3_–PDA@Au-4-MBA are interconnected *via* proportionally increased spike protein-mediated immunoreactions. Quantitative levels of the target SARS-CoV-2 spike protein were evaluated by constructing calibration plots of the log–log transformation of the MINERS signal intensities *versus* spike protein concentration using the characteristic Raman band of 4-MBA at 1058 cm^−1^ shown in Fig. 7c. Our MINERS biosensor's calculated limit of detection (LOD) for SARS-CoV-2 spike protein bioassay in PBS was ∼4.5 fg mL^−1^. Also, SARS-CoV-2 spike protein in cell lysate was detected as a base medium mimicking complex sample matrix (Fig. 7c, blue calibration plot), and the LOD was evaluated as ∼9.7 fg mL^−1^. The representative responses and the LOD results demonstrate the significant contribution or advantage of our MINERS system for detecting SARS-CoV-2 in complex biological samples such as saliva, serum, or nasopharyngeal swabs often collected for COVID-19 screening.

Sensitivity outputs of the SARS-CoV-2 spike protein bioassays achieved by our proposed MINERS technique were possible thanks to the adopted magnetic “pull-down” effect and the specialized MINERS mapping strategy, where data from ∼100 random sites located on solid substrates for the detection were collected, ESI Fig. S9,† showing the robustness and reproducibility MINERS biosensor for SARS-CoV-2 bioassays, ESI Fig. S10.†

Finally, the reliability and selectivity of the proposed MINERS biosensor were demonstrated using several non-target viruses and pseudo-viral particles without observable cross-reactivities, as detailed in ESI Fig. S11.† Overall, the results for the MINERS biosensor showed good spot-to-spot consistency, indicating that this Raman modality *via* MINERS can be used for a viable COVID-19 diagnostic application.

## Conclusions

In this work, we have prepared a multi-functional 3D nanostructure for an advanced application in SERS-based biosensing technology. The design and synthesis route adopted was a facile low-temperature route utilizing a cheaper wet chemistry process. Based on the advanced and synergistic optical properties of the synthesized 3D mag-MoO_3_–PDA@Au NS, a dual-Raman scattering mechanism was promised, followed by a magnetization property for a magnetic “pull-down” effect to engineer a novel MINERS concept towards developing a robust biosensing platform. The proposed MINERS-based biosensor provides not only exceptionally and stably enhanced Raman signals in a magnetic field but also exhibits excellent repeatability and point-to-point consistency in the signals produced. The homogeneity of the MINERS substrates and the absolute uniformity modulated were achieved using an external magnet. With the developed MINERS “hotspot” engineering concept, we have experimentally demonstrated the excellent capability of our innovative multifunctional 3D mag-MoO_3_–PDA@Au NS in the bioassays of pathogens using the SARS-CoV-2 spike protein as a virus model. The MINERS-based biosensing technology can potentially pave the way for detecting the rampaging, rapidly mutating infectious virus for public health surveillance and safety. Importantly, the integration of the MINERS system with a portable-hand-held Raman spectrometer would result in an effective “plug and play” diagnostic option as a tool for combating the COVID-19 pandemic and other future pathogens.

## Experimental

### Materials

All reagents were analytical grade and used without further purification. Ammonium molybdate tetrahydrate, dopamine hydrochloride, ferric chloride, ferrous chloride, and gold chloride were purchased from Aladdin, China. Magnetic NPs and AuNPs were synthesized according to the literature method.^75,76^ Results and discussion of XRD, optical, and SEM characterization of the magnetic NPs and AuNPs are provided in ESI Fig. S12.† The determination of the amount of AuNPs loaded onto the 3D mag-MoO_3_–PDA, as well as the discussion, is detailed in ESI Fig. S13.† Details of all biological materials and agents used in this study can be found in ESI Note 3.†

### Synthesis of mag-MoO_3_–PDA NS

Dopamine hydrochloride 0.3 g and ammonium molybdate tetrahydrate 0.25 g were dissolved in 70 mL of deionized water and stirred at room temperature for 30 min, followed by the addition of 150 mL of absolute ethanol. The reaction mixture was allowed to stir for an additional 10 min, and 0.4 mL of ammonium hydroxide was added to initiate the polymerization reaction. Then, 2 mg mL^−1^ Fe_3_O_4_ NPs were added dropwise to the reaction mixture and left for 2 h. The product was then collected by centrifugation at 5000 rpm for 5 min. The powder product obtained after washing three times with deionized water was dried for further characterization.

### Synthesis of 3D hierarchical mag-MoO_3_–PDA@Au NS

The mag-MoO_3_–PDA NS 5 mg was dissolved in 20 mL of deionized water and stirred at room temperature for 30 min. Then, 2 mg mL^−1^ AuNPs were added dropwise to the solution and left to stir for 3 h. The product was collected by centrifugation and washed several times with deionized water.

### Synthesis of mag-MoO_3_–PDA@Au-4-MBA hybrid

1.5 mL (0.1 g mL^−1^) of mag-MoO_3_–PDA@Au NS was stirred with ethanolic solutions of 4-MBA for 24 h. Then, the resulting product was separated from unbound reagents by washing with ultrapure deionized water by centrifugation at 5000 rpm for 10 min, followed by vacuum drying at 60 °C.

### Synthesis of ACE2-conjugated mag-MoO_3_–PDA@Au-4-MBA

Recombinant ACE-2 protein as a specific binding receptor for SARS-CoV-2 spike protein was conjugated to the surface of mag-MoO_3_–PDA@Au-4-MBA for the capture of SARS-CoV spike protein. By taking advantage of PDA's binding affinity to amine/thiol-containing molecules and protein adsorption,^77^ ACE-2 with amine-containing moieties was chemical adsorbed on the surface of the 3D mag-MoO_3_–PDA@Au NS in alkaline Tris–HCl buffer pH 8.5 according to previously reported protocol.^34,77^ Briefly, 500 μL (10 μg mL^−1^) of ACE-2 (in Tris–HCl buffer, pH 8.5) was added dropwise to 1.5 mL (0.1 g mL^−1^) of mag-MoO_3_–PDA@Au-4-MBA and stirred for 2 h at 4 °C. After the incubation time, a 2% BSA solution was used for blocking. The ACE-2 conjugated mag-MoO_3_–PDA@Au-4-MBA was centrifuged and resuspended in Tris–HCl–0.02% Tween 20 solution and stored at 4 °C for bioassay experiments. ELISA confirmed the conjugation of ACE-2 to the 3D mag-MoO_3_–PDA@Au-4-MBA. A ninhydrin protein assay was used to quantify the conjugated amount of ACE-2 following a protocol that was reported in our previous work.^17^

### MINERS-based detection of SARS-CoV-2 spike protein

Bioassays of SARS-CoV-2 spike protein were performed separately in PBS (pH 7.2) and whole-cell lysate media. To do this, 50 μL of different concentrations of SARS-CoV-2 spike protein (10 fg mL^−1^ to 1 ng mL^−1^) were sequentially incubated with 100 μL of ACE2-mag-MoO_3_–PDA@Au-4-MBA immunocomplex solutions in microtubes for 15 min. With the help of an external magnet, the resulting spike protein-ACE2-mag-MoO_3_–PDA@Au-4-MBA immunocomplexes were enriched and extracted from unbound SARS-CoV-2 spike protein and other matrices. Purified immunocomplexes were re-dissolved in fresh buffer (PBS, pH 7.2) solutions and then deposited on Si substrates for MINERS measurements. The bioassays or selectivity tests of SARS-CoV-2 spike protein in whole-cell lysates followed a similar protocol. The adopted bioassay control experiments were subjected to conditions similar to the detection protocol.

### Confocal Raman measurements

Confocal Raman spectroscopy, SERS, and MINERS analysis were performed on an NRS-7100 onboard microscopic laser Raman spectrophotometer using 532 and 785 nm lasers as excitation sources. Measurements were made using ×100 objective lens at 1% laser power and 2 s integration time. Multi-point MINERS/SERS analyses were performed with a high-speed mapping function from a field of 50 μm to 50 μm with a step size of 2 μm and an exposure time of 2 s. Laser power was 1%, and acquisition time was set to 1 second per pixel. An external bar-shaped NbFeB magnet was commissioned to perform the MINERS measurements. Details of the SERS spectra MoO_3_–PDA and mag-MoO_3_–PDA and the SERS spectra of mag-MoO_3_–PDA@Au excited at 785 nm can be found in the ESI data Fig. S14 and S15.†

### Characterization

The synthesized samples were characterized using a variety of techniques, from electron microscopy to spectroscopy. Scanning electron microscopy (SEM) combined with an energy dispersive X-ray analysis (EDX) were obtained using Zeiss Sigma 500 system (Oxford Instrument). Transmission electron microscopy (TEM) characterization was performed on an America FEI G2 Tecnai operating at 20 kV. X-ray photoelectron spectroscopy (XPS) was obtained using Thermo Fisher Scientific K-Alpha X-ray photoelectron spectrometer with an excitation source of Al Kα = 1486.6 eV. The binding energies were corrected by referencing the C 1s line to 284.80 eV. Powder X-ray diffraction (XRD) patterns were acquired using a Bruker D8 Advance diffractometer with Cu Kα radiation. UV-Vis absorption spectra were acquired with the use of a PerkinElmer-570 spectrophotometer. The magnetic properties of the 3D mag-MoO_3_–PDA@Au NS was measured from −10 kOe to +10 kOe using a superconducting quantum interference device (SQUID magnetometer), Quantum Design, Inc., San Diego, CA, USA.

### Electronic structure calculations

The details of all numerical simulations using the finite element method (FEM) and DFT are provided in ESI Note 4.†

## Author contributions

O. J. A.: conceptualization, investigation, formal analysis, methodology, validation, writing – original draft. N. N.: conceptualization, formal analysis, writing – original draft. D. K. L.: validation, data curation. J. B. L.: resources, E. M. A.: data curation, supervision, writing – review and editing. M. G.: supervision, resources, funding acquisition. E. Y. P.: supervision, writing – review and editing, resources, funding acquisition. All authors have given approval to the final version of the manuscript.

## Conflicts of interest

There are no conflicts to declare.

## Supplementary Material

NA-004-D1NA00746G-s001
