# Supplementary material for: 3D hierarchically porous magnetic molybdenum trioxide@gold nanospheres as a nanogap-enhanced Raman scattering biosensor for SARS-CoV-2
Source: Nanoscale Adv. 2022 Jan 4;4(3):871–83. doi: 10.1039/d1na00746g (PMC9419194; doi:10.1039/d1na00746g)
Supplement: NA-004-D1NA00746G-s001 [file NA-004-D1NA00746G-s001.pdf]

Electronic Supplementary Material (ESI) for Nanoscale Advances.
This journal is © The Royal Society of Chemistry 2022

Supplementary Information

3D hierarchically porous magnetic molybdenum trioxide@gold nanospheres as a nanogap-enhanced Raman scattering biosensor for SARS-CoV-2

Ojodomo J. Achadu[a,†,*], Njemuwa Nwaji[b,†,*], Dongkyu Lee[c], Jaebeom Lee[c], Eser M. Akinoglu[b], Michael Giersig[b,d], Enoch Y. Park[a,e,*]

[a] *Research Institute of Green Science and Technology, Shizuoka University, 836 Ohya Suruga-ku, Shizuoka 422-8529, Japan.*

[b] *International Academy of Optoelectronics at Zhaoqing, South China Normal University, Liyuan Street, 526238 Guangdong, China.*

[c] *Dept. of Chemistry, College of Natural Science, Chungnam National University, 99 Daehak-ro, Yuseong-gu, Daejeon 34134, Korea.*

[d] *Institute of Fundamental Technological Research, Polish Academy of Sciences, 02-106 Warsaw, Poland.*

[e] *Laboratory of Biotechnology, Department of Bioscience, Graduate School of Science and Technology, Shizuoka University, 836 Ohya Suruga-ku, Shizuoka 422-8529, Japan.*

† These authors contributed equally.

[*] Corresponding authors: Research Institute of Green Science and Technology, Shizuoka University, 836 Ohya, Suruga-ku, Shizuoka 422-8529, Japan. Tel (Fax): +81-54-238-4887; park.enoch@shizuoka.ac.jp (E.Y. Park); Tel (Fax): +81-54-238-3306; ojodomo.john.achadu@shizuoka.ac.jp. *International Academy of Optoelectronics at Zhaoqing, South China Normal University, Liyuan Street, 526238 Guangdong. P. R. China.* njemuwa.nwaji@zq-scnu.org (N. Nwaji).

E-mail addresses: ojodomo.john.achadu@shizuoka.ac.jp (O.J. Achadu); njemuwa.nwaji@zq-scnu.org (N. Nwaji); no3_answer@naver.com (D. Lee); nanoleelab@cnu.ac.kr (J. Lee); e.akinoglu@zq-scnu.org (E.M. Akinoglu); giersig@zedat.fu-berlin.de (M. Giersig); park.enoch@shizuoka.ac.jp (E.Y. Park).

Supplementary Note 1: UV-vis properties of 3D mag-MoO_3-PDA@Au NS

UV-vis spectroscopy was used to examine the optical properties of the 3D hierarchical mag-MoO_3-PDA@Au NS. As shown in **Supplementary Figure S6**, strong characteristic broad absorptions are displayed by the mag-MoO_3-PDA@Au NS in the visible and near infrared regions (400 - 800 nm), which is reminiscent of its precursor derivatives of MoO_3-PDA and mag-MoO_3-PDA NS, respectively. These absorptions are probably due to the nanostructures' characteristic localized surface plasmon resonance (LSPR), influenced by the defect states and/or free carrier charge densities of semiconductor MoO_3 and/or plasmonic Au-NPs[1,2]. In addition, the nanostructures have large but thinner hollow surfaces with a high tendency for surface luminosity due to defects and roughness that can be easily used to create local optical or electromagnetic fields[3].

To calculate the free carrier density (*N*) of the 3D mag-MoO_3-PDA@Au NS, the modified Drude model as defined by Eq. S1[2], was employed.

$$N = \frac{\omega_\rho^2 . \varepsilon_0 . m_e}{e^2} \quad \text{(S1)}$$

Where $\boldsymbol{\omega_\rho}$ is the bulk plasmon frequency, $\boldsymbol{\varepsilon_0}$ is the vacuum permittivity, $\boldsymbol{e}$ is the electron charge constant and $\boldsymbol{m_e}$ is the carrier effective mass equal to that of free electrons.

The charge carrier density was estimated to be 5.5×10^{22} cm^{-3}, indicating that mag-MoO_3-PDA@Au NS possesses certain semiconductor-like properties with a significant free carrier density, resulting in the experimentally observed LSPR in visible and NIR regions.

Supplementary Note 2: Calculations of MINERS/SERS enhancement factor (EF)

To evaluate the Raman scattering and enhancement properties of the as-synthesized 3D hierarchical mag-MoO_3-PDA@Au NS, the enhancement factors (EFs) were calculated for both SERS and MINERS modes using the following equation (Eq. S2)[4]:

$$EF = \frac{I_{S(M)}/N_{S(M)}}{I_R/N_R} \qquad \text{(S2)}$$

In this Eq. S2, $I_{S(M)}$ denotes the integrated intensity of the SERS or MINERS mode, such as the aromatic breathing mode of 4-MBA at 1058 cm^{-1} or R6G peak at 65 cm^{-1}. I_R is the intensity of the normal Raman mode of 4-MBA or R6G at the same peaks. $N_{S(M)}$ or N_R are the numbers of the 4-MBA or R6G molecules adsorbed on the 3D hierarchical mag-MoO_3-PDA@Au NS and the numbers of only the 4-MBA or R6G molecules excited at the engaged laser spot.

$N_{S(M)}$ was calculated using Eq. S3:

$$N_{S(M)} = (N_A \, X \, A)/\sigma \qquad \text{(S3)}$$

Where N_A is Avogadro constant, A is effective area occupied by Raman molecules under the laser irradiation, and σ =1.05 × 10^{11} cm^2 mol^{-1} is the per mol area of the molecules' monolayers.

The diameter of the laser spot was determined to be 1.26 or 3.1 μm with the following Eq. S4:

$$D = \left(\frac{\lambda}{NA}\right) X \, 1.22 \qquad \text{(S4)}$$

λ *and* NA are 532 (785 nm) and 0.9 (NA is the Normal function: Magnification X100 of the Raman spectrometer), respectively.

Supplementary Note 3: Biological materials.

Human, recombinant and carrier-free ACE-2 (933-ZN-010), anti-human ACE-2 ectodomain MAb (Clone 171606), SARS-CoV-2 spike protein (D614G variant), SARS-CoV-2 nucleocapsid protein, SARS-CoV-2 spike protein, expressed in 293T whole cell lysate and 293T whole cell lysate were purchased from Funakoshi Co. Ltd (Japan). Human serum (from male AB plasma, USA origin, sterile-filtered) was purchased from Sigma-Aldrich (St. Louis, USA). For selectivity tests, NoV-LPs or HEV-LPs were prepared according to our previous protocol.[5] Clinically isolated influenza virus A/Yokohama/110/2009 (H3N2) was kindly provided by Dr. C. Kawakami of Yokohama City Institute of Health, Japan. Dengue virus cDNA was synthesized by Integrated DNA Technologies (Coralville, Iowa, USA). All experiments and solution preparations were carried out using ultrapure doubly distilled deionized (DI) water (> 18 MΩ·cm) except where otherwise specified. Enzyme-linked immunosorbent assay (ELISA) experiments were performed using a microplate reader (Model 680; Bio-Rad, Hercules, California, USA).

Supplementary Note 4: Computational simulations and DFT calculations

The computational finite element method (FEM) (COMSOL Multiphysics, 5.2a, Boston, MA, USA) was used for all simulations. Electromagnetic wave extinction of MoO_3 or Au models (spherical geometry) were simulated with the FEM method solution of Maxwell's equations. MoO_3 or Au parameters, including refractive index (***n***) and extinction coefficient (***k***) were used according to literature data[6,7] and the materials library in COMSOL Multiphysics software (https://www.comsol.jp/company). Electric field distributions were generated using a plane wave, constant wavelength source at 532 or 785 nm. The electric field output and total enhancements were calculated using: E_{max}^4/E_0^4, where $\boldsymbol{E}_{\mathbf{max}}$ = highest electric field intensity of scattered light and $\boldsymbol{E}_{\mathbf{0}}$ = intensity of incident light electric field.

All DFT calculations were performed with the Quantum Espresso ab-initio code[8]. The generalized gradient approximation was used to treat the electron exchange correlation potential within the Kohn-Sham equation of DFT[9]. The energy cutoff of 29.4 or 44.1 Ry (400 or 600 eV) was set for the real space grids to expand the one-electron wave function, with a 13×13×13 or 13×13×5 k-point meshes based on the unit cell sizes. In all cases, the length of the cell (L) varies from 4 to 10 lattice parameters. Using a conjugate gradient algorithm with residual forces in each component of every atom less than 0.02 eV/Å, all the structures were fully relaxed.

Supplementary Table S1. Enhancement factor (EF) value of Raman reporters (4MBA and R6G) on 3D MoO_3-PDA derivative substrates.

	EF value		
Substrate	MoO_3-PDA	mag-MoO_3-PDA	mag-MoO_3-PDA@Au
4MBA	1.6×10^3	5.4×10^5	7.6×10^9
R6G	6.2×10^3	1.3×10^4	6.5×10^7

Supplementary Table S2. SERS versus MINERS Enhancement of 3D MoO_3-PDA@Au NS in

	3D MoO3-PDA@Au NS	
	SERS (EF)	MINERS (EF)
4MBA	7.6×10^9	2.5×10^{13}
R6G	6.5×10^7	5.2×10^9

the presence of 4MBA and R6G.

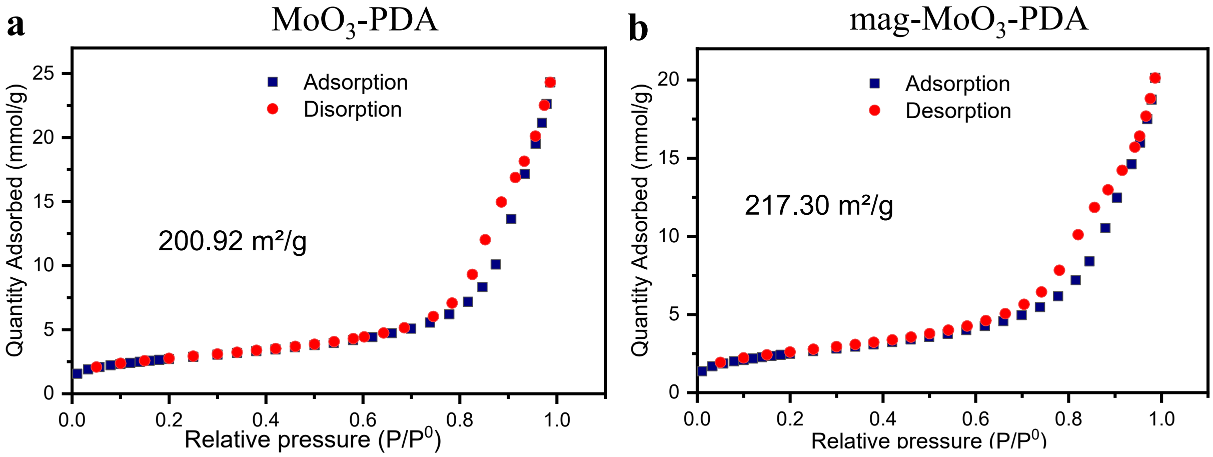

Supplementary Figure. S1. N_2 sorption of the nanostructures (a) MoO_3-PDA, and (b) mag-MoO_3-PDA, showing their specific surface areas.

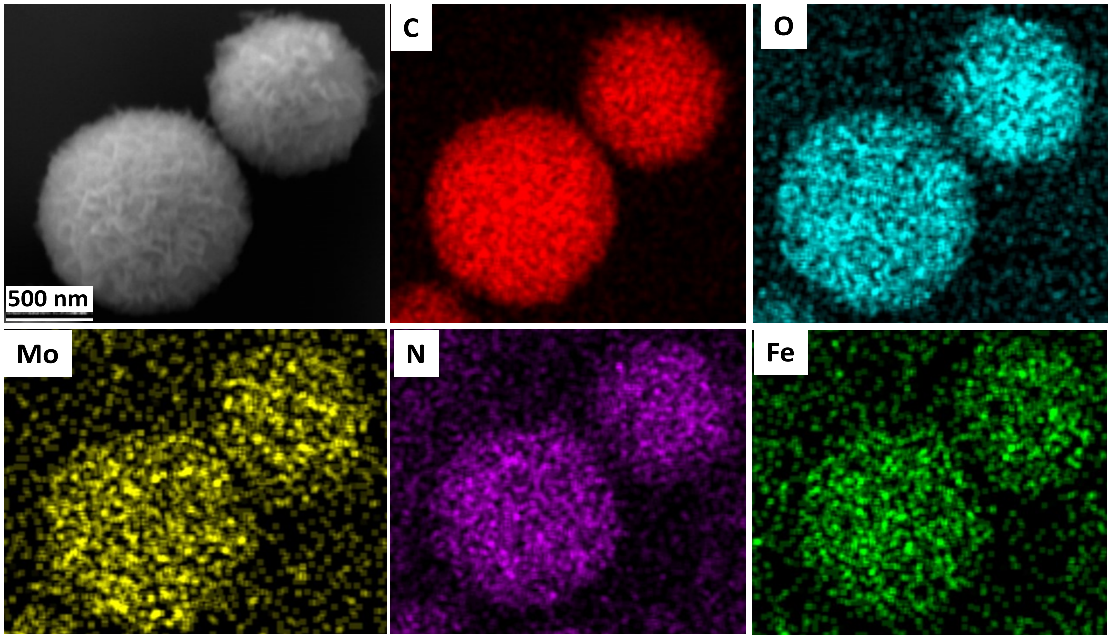

Supplementary Figure S2. EDS mapping of mag-MoO_3-PDA NS, showing the various elemental compositions of Mo, C, N, O and Fe, respectively.

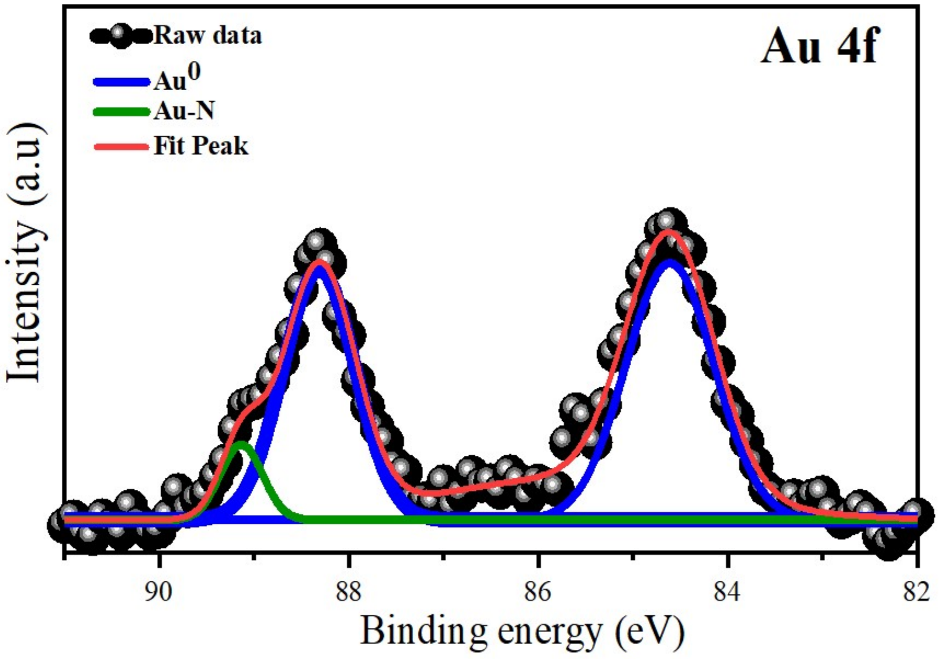

Supplementary Figure S3. High resolution XPS spectra Au 4f of mag-MoO_3-PDA@Au NS.

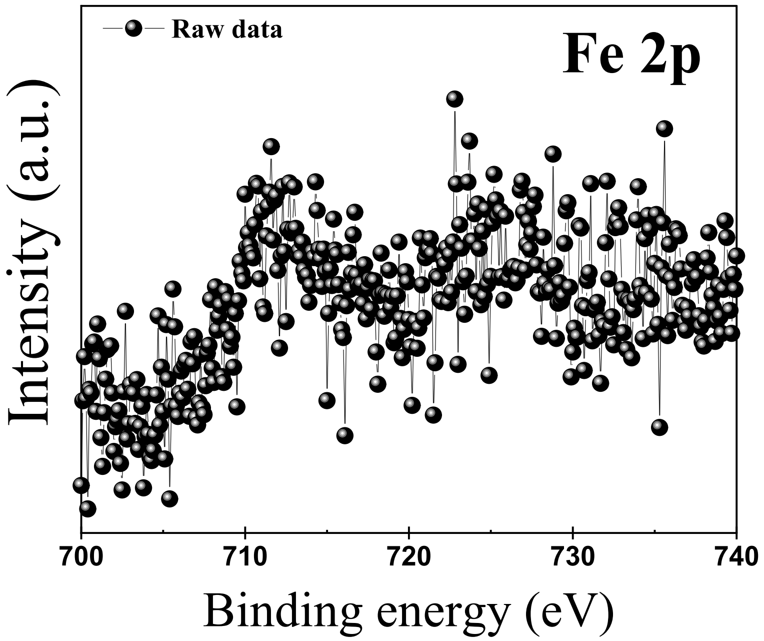

Supplementary Figure S4. High resolution XPS spectra of Fe 2p of mag-MoO_3-PDA@Au NS.

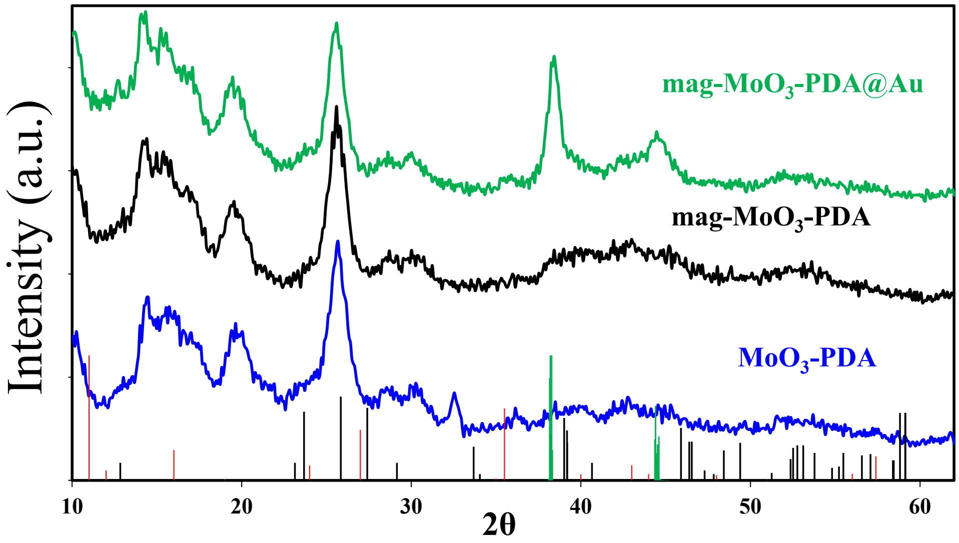

Supplementary Figure S5. XRD data of MoO_3-PDA nanostructure and its magnetic or Au NPs derivatives. Vertical line represents the standard diffraction pattern for MoO_3 (back, JCPDS No. 05-0508), Fe_3O_4 (red, JCPDS No. 89-0691 and AuNPs (green).

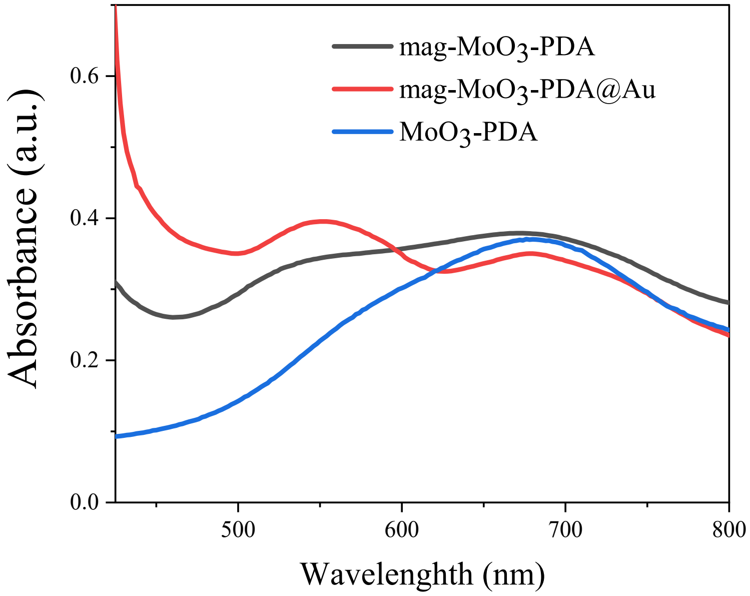

Supplementary Figure S6. UV-vis spectra of MoO_3-PDA nanostructure and its magnetic (mag-MoO_3-PDA) or Au NPs functionalized (mag-MoO_3-PDA@Au NS) derivatives.

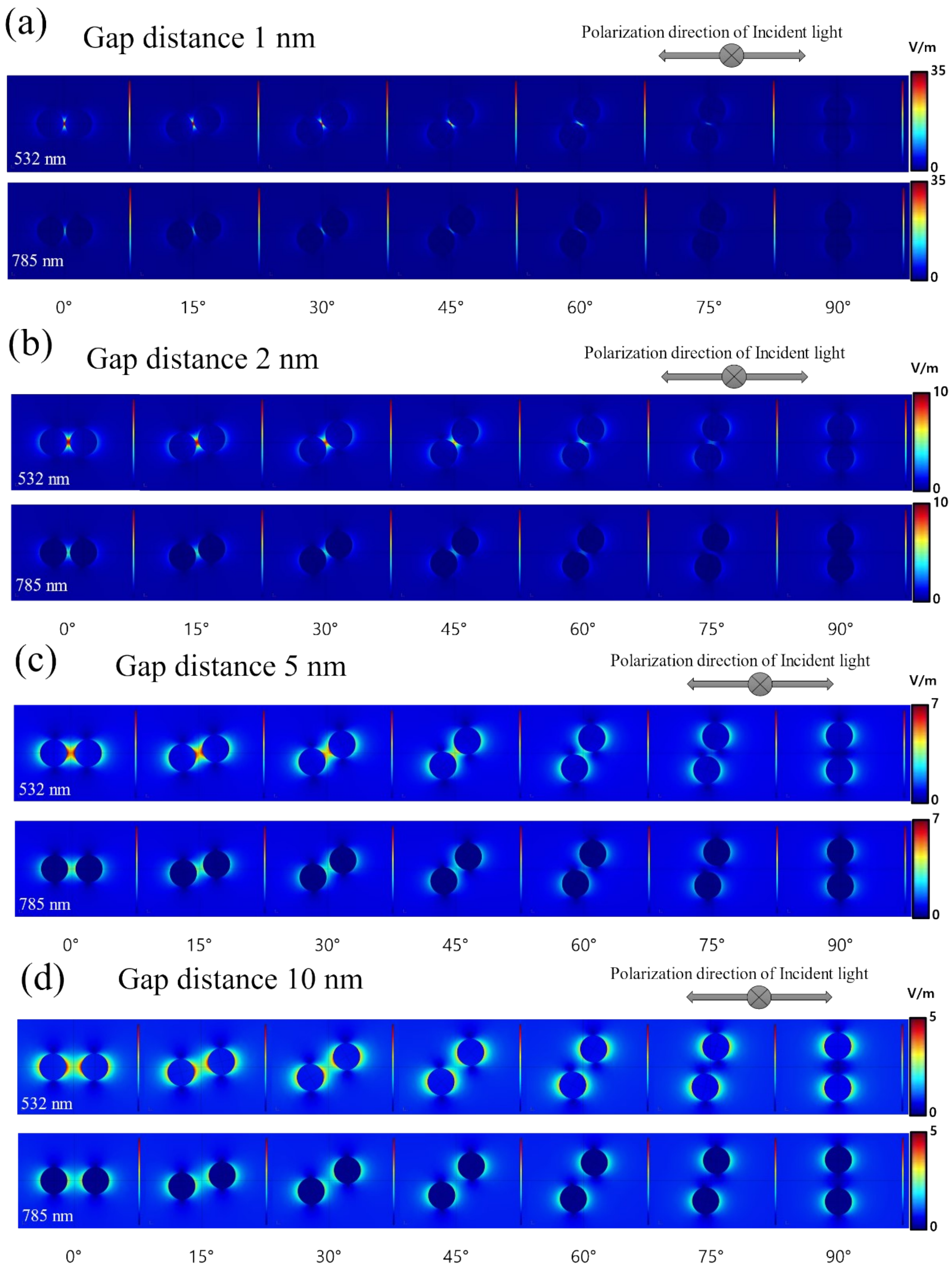

Suppl

ementary Figure S7. E-field distribution (in air medium, λ = 532 or 785 nm, E_0 = 1 V/m) at nanogaps of 1 nm (**a**), 2 nm **(b**), 5 nm (**c**) and 10 nm (**d**), respectively, in the approximate

dimeric 3D mag-MoO_3-PDA@Au nanostructures using finite element method (FEM) numerical simulations and theoretical and calculations.

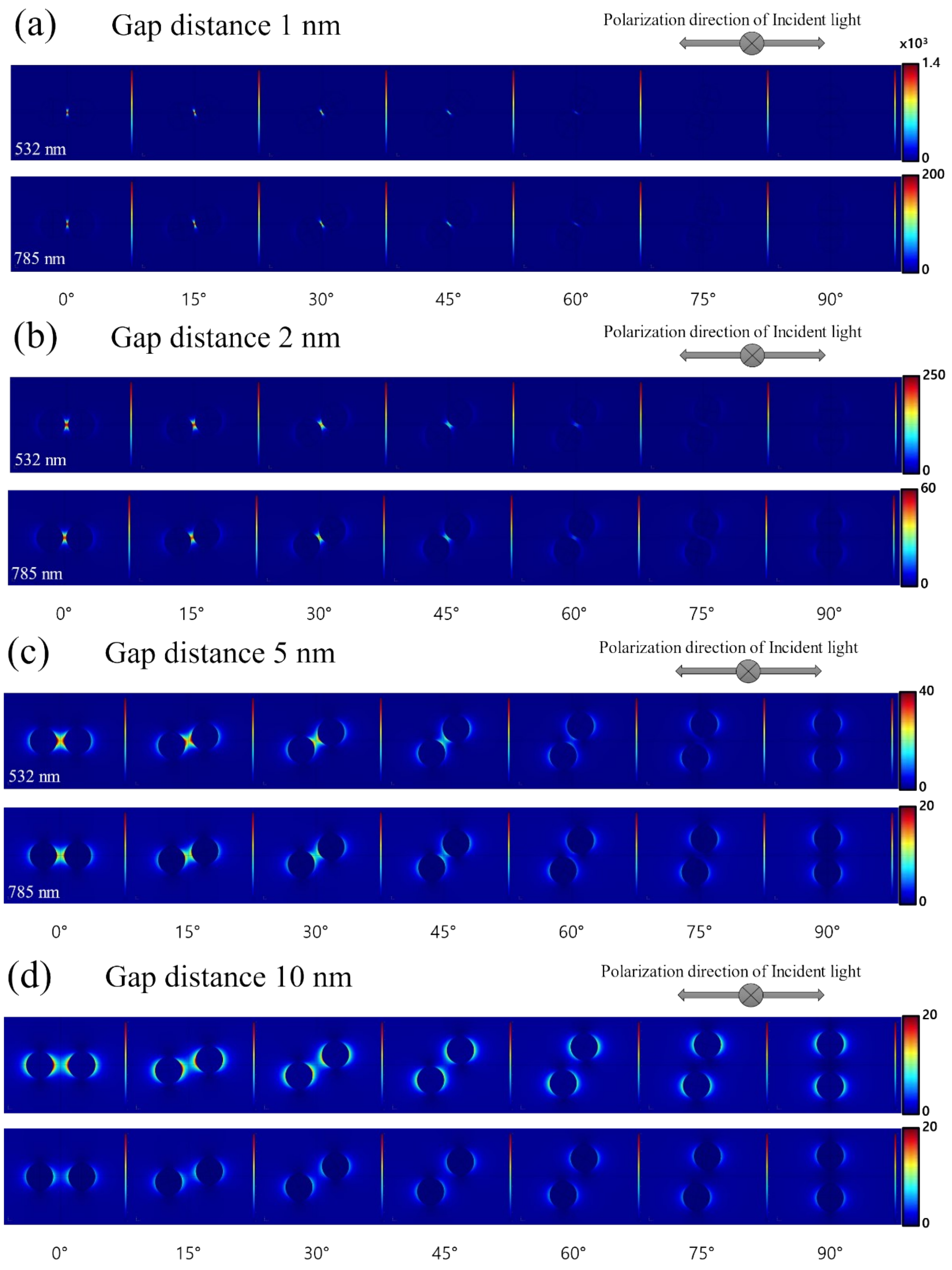

Supplementary Figure S8. Electromagnetic field enhancements simulations (in air medium, λ = 532 or 785 nm, E_0 = 1 V/m) at nanogaps of 1 nm (**a**), 2 nm (**b**), 5 nm (**c**) and 10 nm (**d**), respectively, in the approximate dimeric 3D mag-MoO_3-PDA@Au nanostructures using finite element method (FEM).

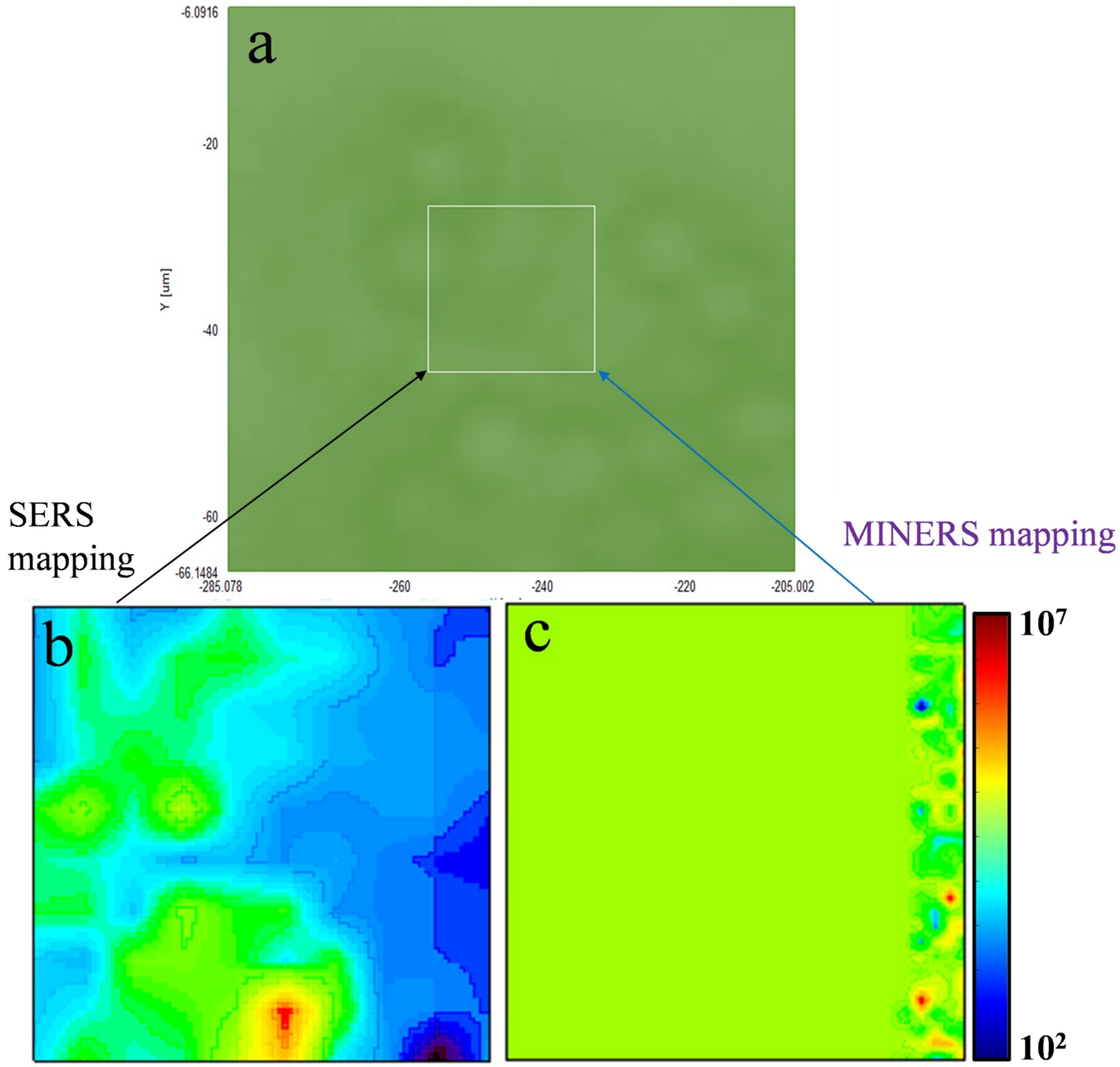

Supplementary Figure S9. (**a**) Optical photograph of SARS-CoV-2 spike protein detection substrate at 1 ng mL^{-1} of spike protein interaction with ACE2-mag-MoO_3-PDA@Au-4MBA immunocomplex and the corresponding mapping results for conventional SERS (**b**), and our proposed MINERS at a magnetic field intensity of 150 mT (**c**).

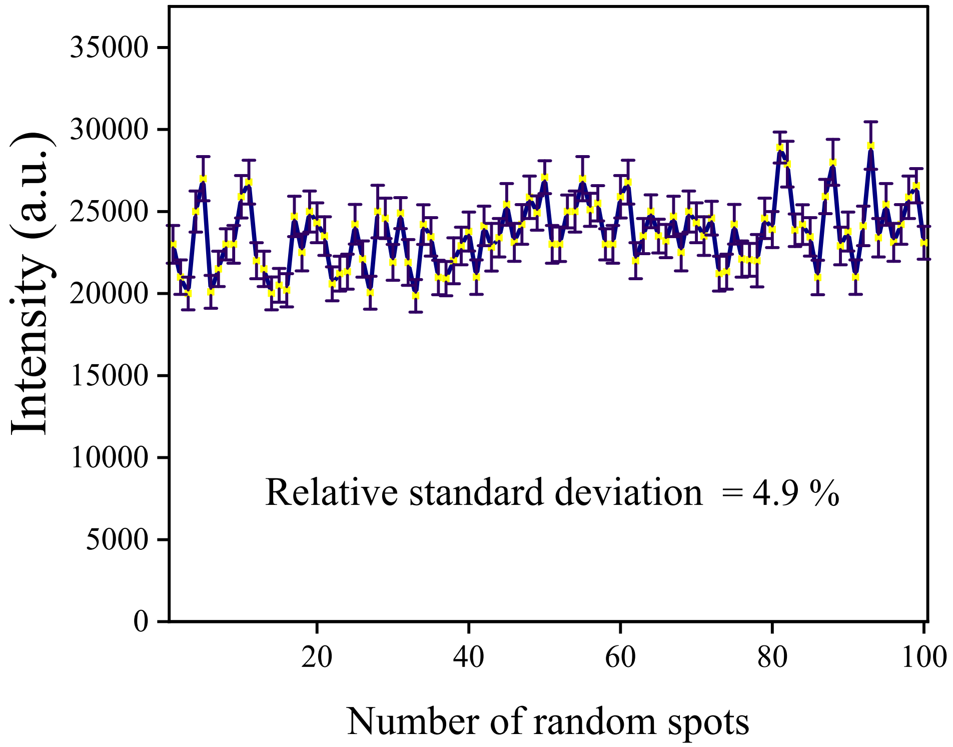

Supplementary Figure S10. Showing the randomly mapped spots (100) as a proof of the robust and for reproducible detection of SARS-CoV-2 spike protein using MINERS biosensor. The calculated relative standard deviation (RSD) of <5 % demonstrate the impressive spot-spot variation of our MINERS strategy.

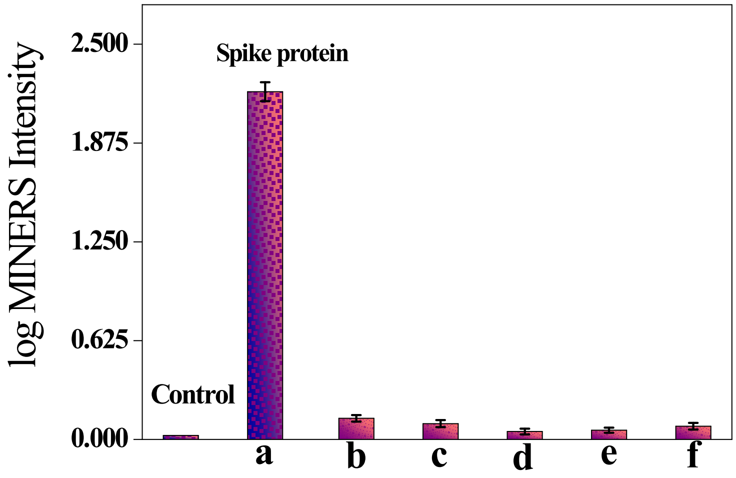

Supplementary Figure S11. Selective detection of target SARS-CoV-2 Spike protein (**a**) compared other non-target virus and proteins such as SARS-CoV-2 Nucleocapsid protein (**b**), Hepatitis E virus-like particles (HEV-LPs) (**c**), Influenza virus A/H3N2 (**d**), Bovine serum albumin (**e**) and Noro virus-like particles (NoV-LPs) (**f**). Concentration of spike protein (**a**) = 1 $ngmL^{-1}$; **b**, **c**, **e** and **f** = 5 μgmL^{-1} and **d** = 5 x 10^4 $copymL^{-1}$. Control = blank (PBS).

Supplementary Note 5: AuNPs and magnetic NPs Characterization data

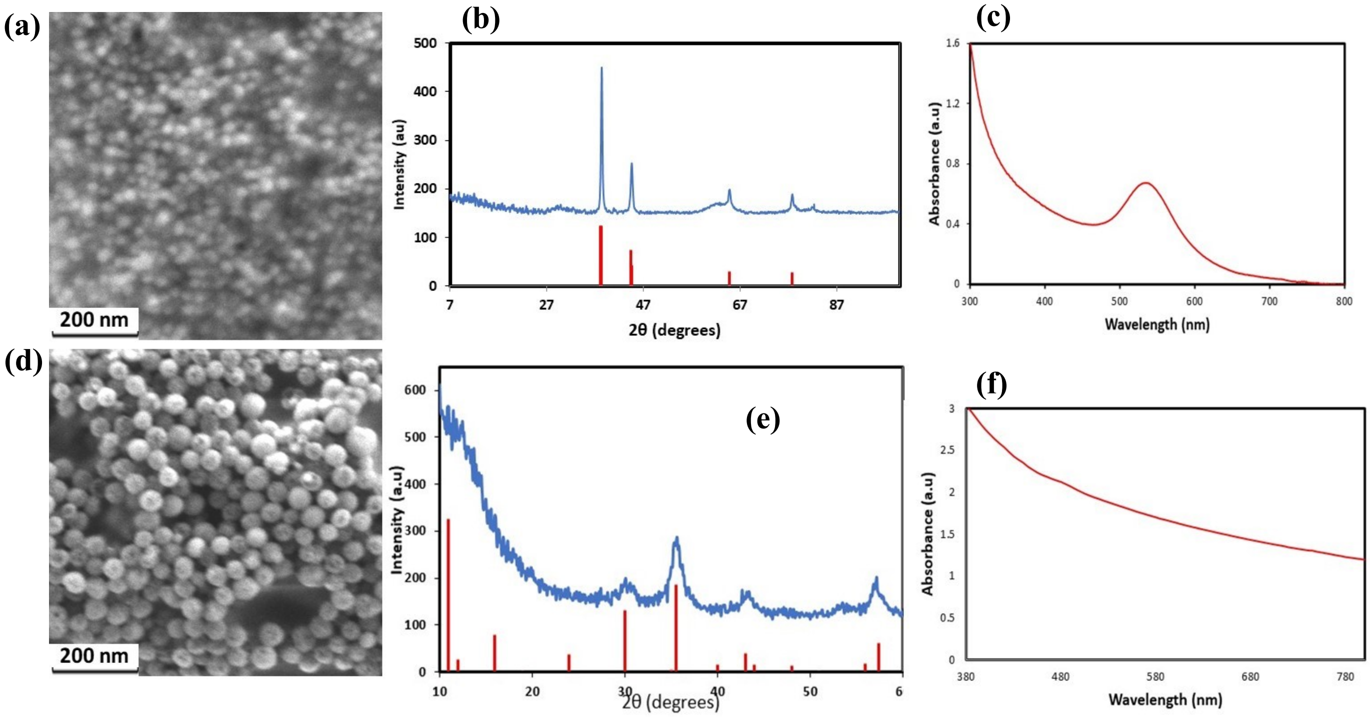

Supplementary Figure S12. AuNPs characterization. (**a**) SEM, (**b**) XRD and (**c**) UV-vis. Magnetic NPs characterization (**d**) SEM, (**e**) XRD and (**f**) UV-vis.

Supplementary Note 6: Au NPs loading on mag-MoO3-PDA NS

The percentage of AuNPs loaded onto the 3D mag-MoO_3-PDA was investigated following literature methods[10]. This involves comparing the Q band absorbance intensity of the AuNPs before loading and the supernatant solution after loading (**Figure S13**). Using Beer-Lambert law and molar extinction coefficient for 20 nm AuNPs[11]. The amount of AuNPs loaded onto the 3D mag-MoO_3-PDA was evaluated to be ~27.6%.

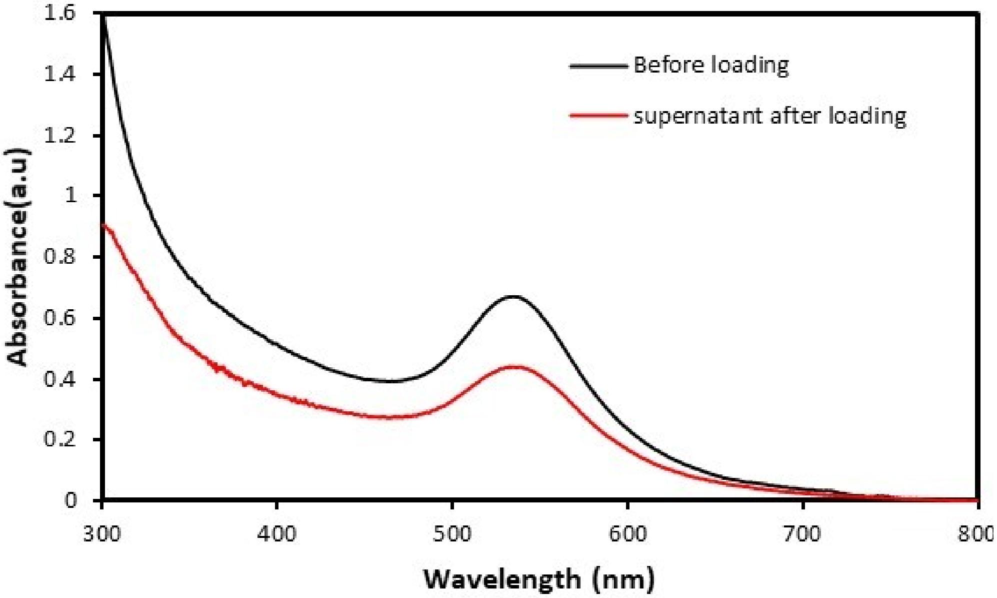

Supplementary Figure S13: UV-Vis absorption change before and after loading AuNPs onto 3D mag-MoO_3-PDA.

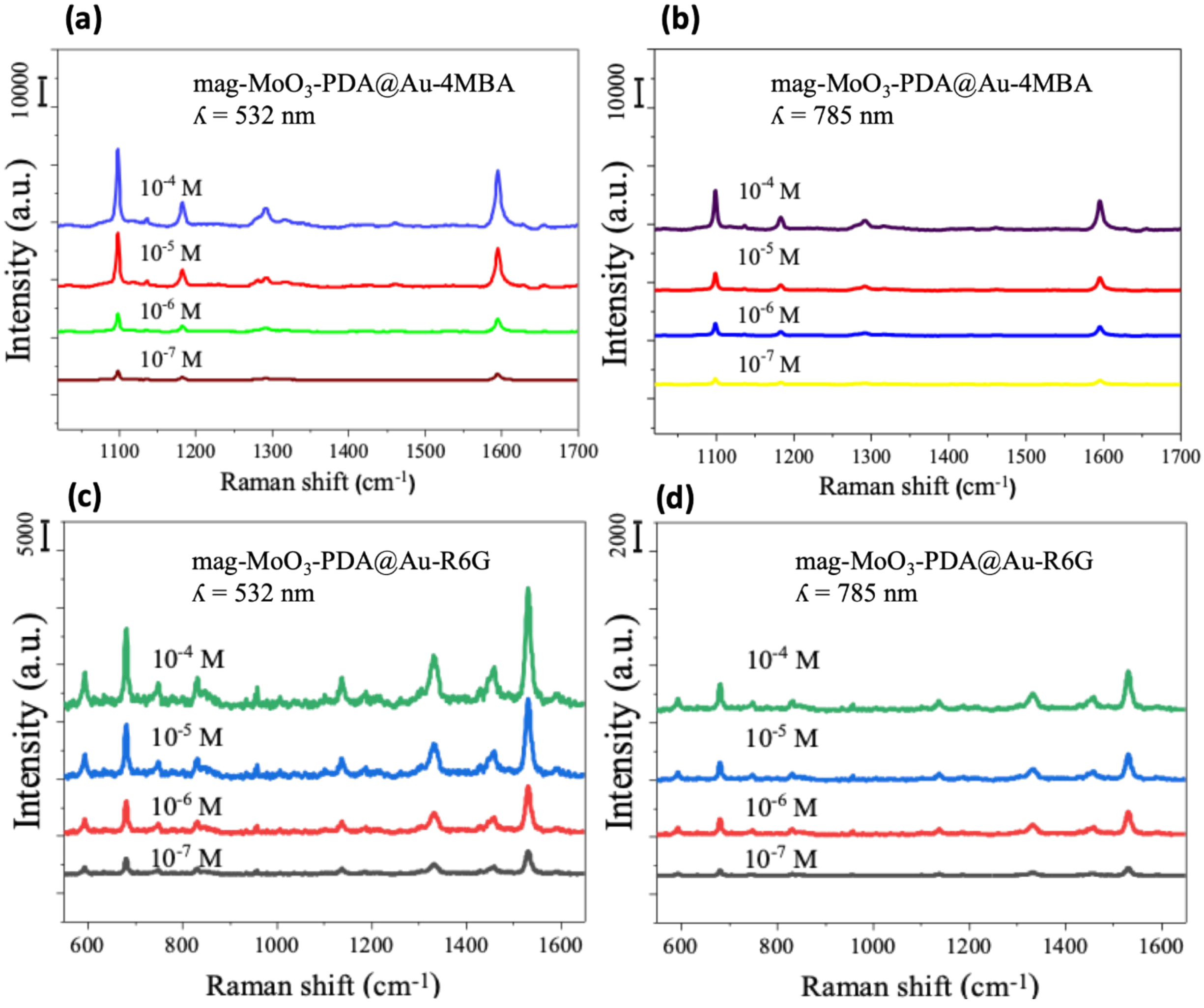

Supplementary Figure S14: SERS spectra of the 3D mag-MoO_3-PDA@Au in the presence of different concentrations of 4-MBA and R6G at 532 nm (**a**, **c**) and 785 nm (**b**, **d**).

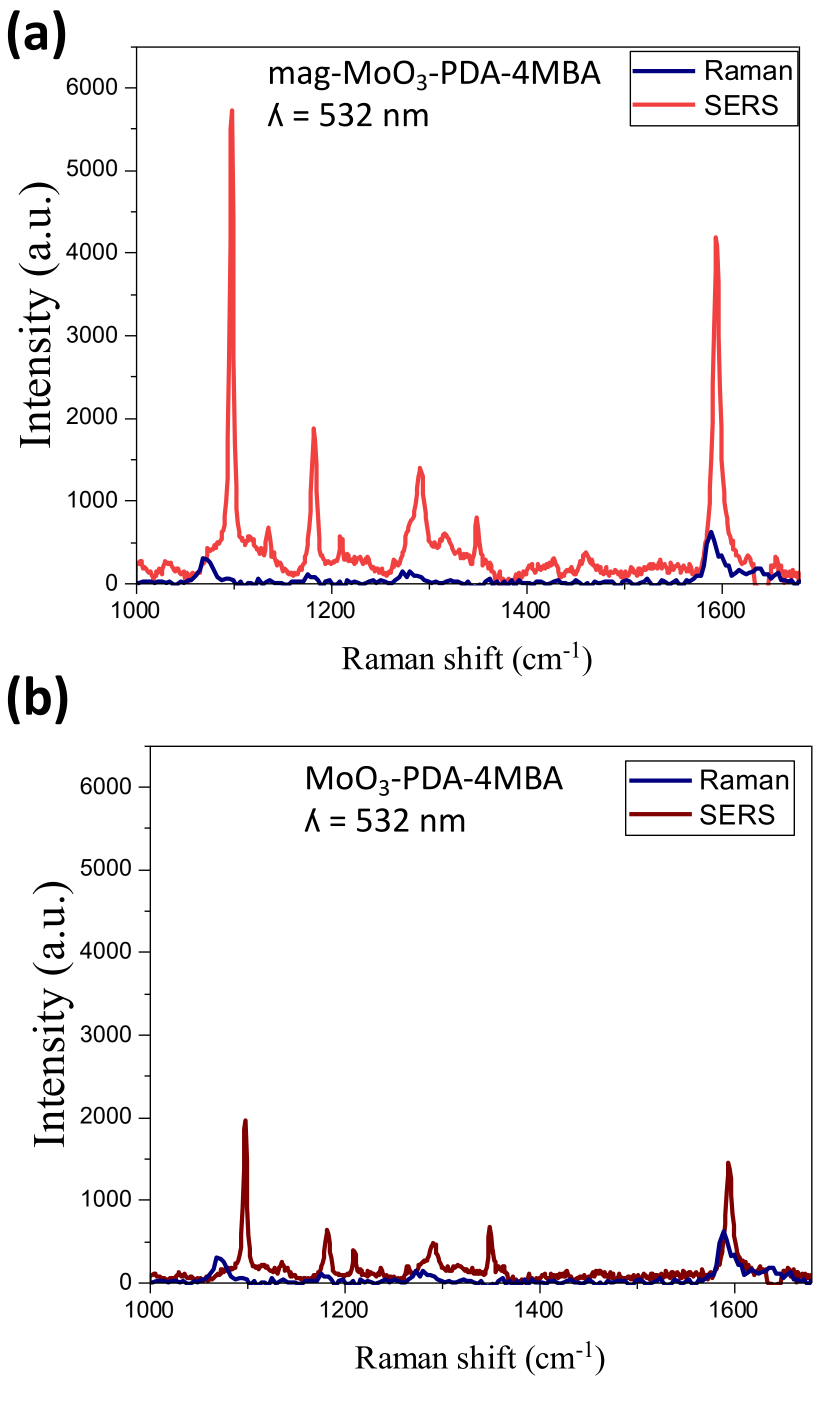

Supplementary Figure S15: SERS spectra of the mag-MoO_3-PDA (**a**) and MoO_3-PDA (**b**) in the presence of 4-MBA at 532 nm excitation.

Supplementary References

1. White, R. T.; Thibau, E. S.; Lu, Z.H. Interface Structure of MoO_3 on Organic Semiconductors. *Sci Rep*. **2016**, 6, 21109.
2. Kheirandish, A.; Javan, S.; Mohammadzadeh, H.; Modified Drude model for small gold nanoparticles surface plasmon resonance based on the role of classical confinement. *Sci Rep.* **2020**, 10, 6517.
3. Shvalya, V.; Filipič, G.; Zavašnik, J.; Abdulhalim, I.; Cvelbar, U. Surface-enhanced Raman spectroscopy for chemical and biological sensing using nanoplasmonics: The relevance of interparticle spacing and surface morphology. *Appl. Phys. Rev* **2020**,7, 31307.
4. Zhou, L.; Zhou, J.; Lai, W.; Yang, X.; Meng, J.; Su, L.; Gu, C.; Jiang, T.; Pun, E. Y. B.; Shao, L.; Petti, L.; Sun, X. W.; Jia, Z.; Li, Q.; Han, J.; Mormile, P. Irreversible accumulated SERS behavior of the molecule-linked silver and silver-doped titanium dioxide hybrid system. *Nat Commun.* **2020**, 11, 1785.
5. Ahmed, S. R.; Takemura, K.; Li, T. C.; Kitamoto, N.; Tanaka, T.; Suzuki, T.; Park, E. Y. Size-controlled preparation of peroxidase-like graphene-gold nanoparticle hybrids for the visible detection of norovirus-like particles. *Biosens. Bioelectron.* **2017**, 87, 558–565.
6. Achadu, O. J.; Abe, F.; Li, T.C.; Khoris, I.M.; Lee, D.K.; Lee, J.; Suzuki, T.; Park, E. Y. Molybdenum Trioxide Quantum Dot-Encapsulated Nanogels for Virus Detection by Surface-Enhanced Raman Scattering on a 2D Substrate. *ACS Appl. Mater. Interfaces* **2021**, 13, 27836–27844.
7. Lajaunie, L.; Boucher, F.; Dessapt, R.; P. Moreau. Strong anisotropic influence of local-field effects on the dielectric response of α-MoO3. *Phys. Rev. B*. **2013**, 88, 115141.

8. Giannozzi P.; Baroni S.; Bonini N.; Calandra M.; Car R.; Cavazzoni C. QUANTUM ESPRESSO: a modular and open-source software project for quantum simulations of materials. *J. Phys. Condens. Matter.* **2009**, 21, 395502.
9. Perdew J. P.; Burke, K.; Ernzerhof M. Generalized Gradient Approximation Made Simple. *Phys. Rev. Lett.* **1996**, 77, 3865.
10. Li, J.-F. Zhao, N. Won, H. Jin, S. Kim, J.-Y. Chen, Quantum Dot – Aluminum phthalocyanine Conjugates perform photodynamic reactions to kill cancer cells via fluorescence resonance energy transfer (FRET), Nanoscale Res. Lett. 2012, **12**, 386.
11. Xiong, L.; Atwater, M.; Wang, J.; Huo Q. Extinction coefficient of gold nanoparticles with different sizes and different capping ligands. Colloids Surf B Biointerfaces 2007, **58**, 3.
